# Dysregulated endothelial cell markers in systemic lupus erythematosus: a systematic review and meta-analysis

**DOI:** 10.1186/s12950-023-00342-1

**Published:** 2023-05-16

**Authors:** S. C. Bergkamp, M. J. Wahadat, A. Salah, T. W. Kuijpers, V. Smith, S. W. Tas, J. M. van den Berg, S. Kamphuis, D. Schonenberg-Meinema

**Affiliations:** 1 Department of Paediatric Immunology, Rheumatology and Infectious Diseases, Emma Children’s Hospital, Amsterdam University Medical Centres (AUMC), University of Amsterdam, Meibergdreef 9, 1105 AZ Amsterdam, The Netherlands; 2 Department of Paediatric Rheumatology, Sophia Children’s Hospital, Erasmus University Medical Centre, Rotterdam, The Netherlands; 3 Department of Immunology, Erasmus University Medical Center, Rotterdam, The Netherlands; 4 Department of Internal Medicine, Ghent University, Ghent, Belgium; 5 Department of Rheumatology, Ghent University Hospital, Ghent, Belgium; 6 Unit for Molecular Immunology and Inflammation, VIB Inflammation Research Centre (IRC), Ghent, Belgium; 7 Department of Rheumatology and Clinical Immunology, and Laboratory for Experimental Immunology, Amsterdam Rheumatology and Immunology Centre, Amsterdam University Medical Centres (AUMC), University of Amsterdam, Amsterdam, The Netherlands

**Keywords:** Endothelial cell, Endothelial cell markers, Systemic lupus erythematosus, Premature atherosclerosis, Cardiovascular disease

## Abstract

**Objectives:**

To perform a systematic literature review and meta-analysis on endothelial cell (EC) markers that are involved and dysregulated in systemic lupus erythematosus (SLE) in relation to disease activity, as EC dysregulation plays a major role in the development of premature atherosclerosis in SLE.

**Methods:**

Search terms were entered into Embase, MEDLINE, Web of Science, Google Scholar and Cochrane. Inclusion criteria were 1) studies published after 2000 reporting measurements of EC markers in serum and/or plasma of SLE patients (diagnosed according to ACR/SLICC criteria), 2) English language peer reviewed articles, and 3) disease activity measurement. For meta-analysis calculations, the Meta-Essentials tool by Erasmus Research Institute and of Management (ERIM) was used. Only those EC markers, which were 1) reported in at least two articles and 2) reported a correlation coefficient (i.e. Spearman’s rank or Pearson’s) between the measured levels of the EC marker and disease activity were included. For meta-analyses, a fixed effect model was used.

**Results:**

From 2133 hits, 123 eligible articles were selected. The identified SLE-related endothelial markers were involved in EC activation, EC apoptosis, disturbed angiogenesis, defective vascular tone control, immune dysregulation and coagulopathy. Meta-analyses of primarily cross-sectional studies showed significant associations between marker levels and disease activity for the following endothelial markers: Pentraxin-3, Thrombomodulin, VEGF, VCAM-1, ICAM-1, IP-10 and MCP-1. Dysregulated EC markers without associations with disease activity were: Angiopoeitin-2, vWF, P-Selectin, TWEAK and E-Selectin.

**Conclusions:**

We provide a complete literature overview for dysregulated EC markers in SLE comprising a wide range of different EC functions. SLE-induced EC marker dysregulation was seen with, but also without, association with disease activity. This study provides some clarity in the eminent complex field of EC markers as biomarkers for SLE. Longitudinal data on EC markers in SLE are now needed to guide us more in unravelling the pathophysiology of premature atherosclerosis and cardiovascular events in SLE patients.

**Supplementary Information:**

The online version contains supplementary material available at 10.1186/s12950-023-00342-1.

## Introduction

Systemic lupus erythematosus (SLE) is a severe, lifelong autoimmune disease known for its heterogeneous presentation, disease flares and multi-system organ involvement. In general, patients with inflammatory diseases have an increased risk of developing atherosclerosis, the predominant cause of cardiovascular disease (CVD) [1, 2]. Compared to other inflammatory diseases, SLE patients are known to be at high risk for premature atherosclerosis [3, 4] and the majority of SLE-associated deaths have been attributed to cardiovascular disease (CVD) [5]. The high prevalence of CVD in SLE is explained by both traditional risk factors (e.g. obesity, hypertension) and SLE-specific risk factors, such as corticosteroid treatment, renal impairment and presence of antiphospholipid antibodies [6]. In case–control studies, the risk of developing CVD is increased up to 17-fold for SLE patients compared to healthy age-matched controls. In female SLE patients between the age of 35–44 years, this risk is even up to 50 times higher [7]. Conrad et al. endorsed the high premature cardiovascular risk in SLE patients in their study with a large cohort (> 10.000 SLE patients) [8]. With 90% of the SLE patients being female, this is an important risk to take into account when caring for female patients with SLE, specifically since CVD is more prevalent in males in the general population [9]. Indeed, Vogel et al. emphasized that women with CVD in general remain understudied and undertreated [10]. Age of disease onset is also linked to SLE-associated premature atherosclerosis, as CVD manifests at a much younger age in childhood-onset SLE patients (cSLE) when compared to adult-onset SLE patients. Cardio- and cerebrovascular complications have been reported for 5–10% of young adults with cSLE, with the majority of events occurring between the age of 20–40 [11]. These findings in SLE are worrisome, especially when considering that multiple studies only include survivors of cardiovascular events.

The pathophysiologic mechanisms underlying premature atherosclerosis in SLE are complex and not completely understood [12]. In this process, endothelial cells (ECs), that form the vascular endothelium, play a major role. In autoimmune diseases like SLE, EC promote chronic inflammation through various processes such as angiogenesis, attraction of immune cells, and antigen presentation [13]. Chronic inflammation in SLE augments the production of reactive oxygen species, partially through antiphospholipid (aPL) autoantibodies [14]. aPL autoantibodies can directly activate monocytes and consecutively, these monocytes interact with the endothelium. The aPL antibodies also cross-react with oxidized low-density lipoproteins. As a result, low-density lipoprotein (LDL) gets oxidized and this induces activation of ECs. These events are generally considered to form the initial stage of endothelial dysfunction and atherogenesis in SLE patients [15–17]. Next, activation of ECs triggers the production and release of pro-inflammatory cytokines such as monocyte chemoattractant protein 1 (MCP-1), interleukin-6 (IL-6) and tumour-necrosis factor α (TNF-α) [18, 19]. This induces EC overexpression of surface molecules on the vascular wall that promote the adhesion (vascular cell adhesion molecule 1 (VCAM-1) and intercellular cell adhesion molecule 1 (ICAM-1)), rolling (selectins) and attachment (integrins) of monocytes [18]. Subsequently, MCP-1-mediated transmigration of monocytes into the arterial intima occurs, enabling these leukocytes to differentiate into macrophages. In turn, stimulation by multiple factors including TNF-α, interleukin-1 (IL-1) and homocysteine as well as phagocytosis of oxidized LDL (oxLDL) causes macrophages to develop into lipid-rich foam cells. These foam cells then form the basis of the growing atherosclerotic lesion on the arterial lumen. The abundantly present inflammatory cytokines drive new smooth muscle cells to migrate towards the atherosclerotic lesion and enable the macrophages’ derived foam cells to further proliferate, thereby causing the lesion to expand and facilitating plaque-generation. Simultaneously, augmented dendritic cell production of interferon α (IFN-α) induces apoptosis of endothelial precursor cells (EPC) and circulating angiogenic cells, and hampers differentiation of these cells to mature ECs. Consequently, the secretion of IFN-α, induces the EPC apoptosis and converts circulating angiogenic cells (CAC’s) to dendritic cells. This leads to lower ability of of CAC’s to repair vascular damage from the endothelium [19]. In addition, aPL antibodies interact with ECs and monocytes, leading to a procoagulant phenotype and enhancing the risk of thrombosis [20]. With all of this above in mind, the endothelium in SLE suffers from inflammation, defective repair and pro-thrombogenic factors.

Recently, we have observed high numbers of capillary haemorrhages by nailfold capillaroscopy in our paediatric cohort of SLE [21, 22]. These capillary haemorrhages were correlated with SLEDAI and nephritis and might be reflecting of endothelial damage [21, 22]. When SLE-induced atherosclerosis manifests as CVD-related clinical events (e.g. myocardial infarction), vascular damage is already advanced and often irreversible [15]. Hence, early-stage detection of atherosclerosis and vascular damage is highly desirable. Yet, screening protocols for detecting biomarkers that predict atherosclerotic risks are not current practice in clinical care of SLE-patients, partially because it is not clear which biomarkers can be used for this screening. As described above, a growing body of evidence indicates a complex but central role of EC dysfunction in the development of accelerated atherosclerosis in SLE. We defined EC markers as proteins expressed and/or produced by ECs, which facilitate interactions between ECs or between ECs and immune cells, and sometimes can activate ECs in specific relation to SLE. SLE-related EC markers hold promise to be valuable biomarkers for premature atherosclerosis in SLE, as several appear to be differentially expressed and/or secreted upon EC dysfunction. This systematic review and meta-analysis aims to identify the specific EC markers that are dysregulated in SLE and to investigate possible associations of these dysregulated EC markers with disease activity.

## Methods

### Search strategy and selection

**Fig. 1:**
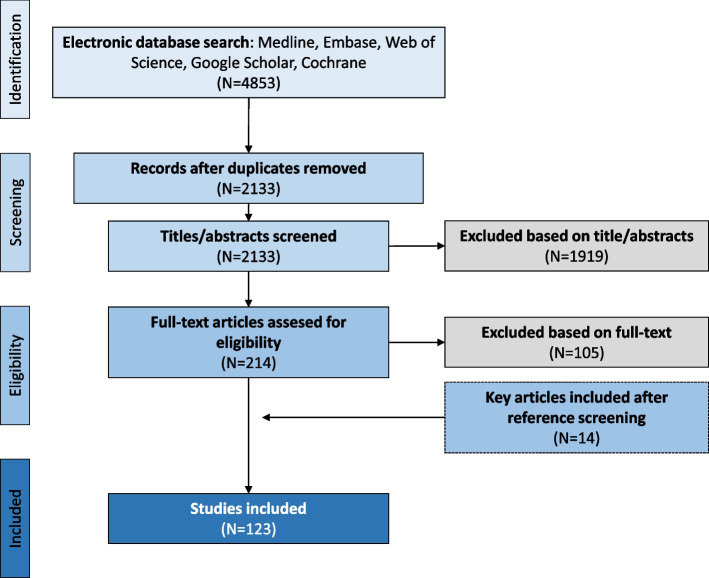
Flow chart. Flow chart reflecting the selection process of articles following the PRISMA-P guidelines

The search for this systematic literature review was performed according to the Preferred Reporting Items for Systematic review and Meta-analysis Protocols (PRISMA-P) 2015 [23]. The PRISMA 2020 guideline was not yet published at time of start of the systematic review. (PRISMA 2020 Guideline was published in March 2021). In July 2020, the search terms were entered in Embase, MEDLINE, Web of Science, Google Scholar and Cochrane. Search terms and search strings per database can be found in Supplementary File 1. After removal of duplicates, selection of articles eligible for full text screening was based upon title and/or abstract. In April 2022, an update of the search was conducted. The following inclusion criteria for eligibility were used: 1) published studies after the year 2000 that reported measurement of EC markers in serum and/or plasma of SLE patients diagnosed according to ACR/SLICC criteria, 2) English language peer reviewed articles and 3) measurement of disease activity with a validated SLE disease activity index (i.e. SLEDAI, BILAG, SLAM, ECLAM or PGA). Exclusion criteria were 1) case reports or editorials, 2) studies performed in animals and 3) studies with microRNA biomarkers*.* Figure 1 summarizes the results of the screening process.

Two reviewers (S.B. and M.W.) independently screened all titles and abstracts for eligibility. If there were discrepancies in eligibility between SB and M.W., consensus was reached by adding a third reviewer (D.S.) to make the final decision for inclusion or exclusion. The selected articles were read as full text by all three reviewers and the list of references of these articles was screened for additional eligible articles.

### Quality assessment

The included full-text articles underwent quality appraisal by SB, M.W., and DS, using a standardized scoring sheet from the National Institutes of Health (NIH) Quality Assessment tool for Observational Cohort and Cross Sectional Studies [24]. This contains at least 12 and maximal 14 questions (depending on study design), resulting in a score ranging between 0–14 (see Supplementary File 3). In a second phase, SB, MW and DS reached consensus on the scores. No studies were excluded based on their quality.

### Data extraction

Data regarding SLE-related EC markers were extracted from the articles using a predefined data extraction form. Supplementary File 2 provides an overview of extracted data from all included studies in chronological order (e.g. authors, year, studied markers, study design, number of patients, age patients, disease duration, number of controls, serum/plasma, disease activity score, presence or absence of associations with disease activity, method of blood analysis (ELISA/Flow cytometry/Luminex)).

### Meta-analyses

For meta-analyses, only those EC markers, which were 1) reported in at least two articles and 2) reported a correlation coefficient (i.e. Spearman’s rank or Pearson’s) between the measured levels of the EC marker and disease activity were included. Separate meta-analyses for the markers with Spearman’s rho as well for Pearson’s r correlations were performed (if possible, i.e. at least two studies with the same correlation measure). In the meta-analysis for a specific marker, we used Fisher’s Z transformation to identify the overall size effect based on the sample size and Spearman’s rank or Pearson’s correlation coefficients of the individual studies, known as the Hedges and Olkin method [25]. For calculations, the Meta-Essentials tool by Erasmus Research Institute and of Management (ERIM) was used [26]. We used a fixed-effects model for the meta-analyses, due to the low number of studies. Additionally, the Meta-Essentials tools uses the ‘weighted variance method’ for the calculation of a confidence interval (CI) for the overall correlation coefficient [27]. This ‘weighted variance method’ is based on a t distribution, with k (degrees of freedom = n (amount of studies)—1). In the current work, overall correlation coefficients were considered statistically significant when the 95% CI did not include ‘0’ and t-distribution-derived *p*-value was < 0.05. To assess meta-bias, Egger's test, along with a funnel plot and Begg’s test will be used to check for publication bias.

Forest plots and the Ferris Wheel Plot are generated using GraphPad Prism 9 Adobe Illustrator 2020, respectively.

## Results

From the searched databases 4853 articles were retrieved. After removal of 2133 duplicates, we excluded 1919 articles based on title and abstract. We obtained 214 full-text papers for further evaluation and to identify possibly overlapping study populations. By cross-referencing retrieved papers, we identified an additional 14 papers. A total number of 105 papers was subsequently excluded after full-text evaluation. Three of them had overlapping study populations or data. Finally, we included 123 studies that met the inclusion criteria (Fig. 1). The majority of the studies (90%) had a cross-sectional design, 10% of the studies used a longitudinal design (*n* = 12, mean of 32.3 patients). The age of patients across the different studies ranged from 8 to 77 years, but few studies included patients < 18 years of age (*n* = 6/123 studies (4.9%), with a mean of 48.2 patients) [28–33]. For a slight majority (55,6%) of the studies, disease duration at time of sample collection was mentioned. Further details of all included studies are reported in Supplementary File 2 and quality assessments are reported in Supplementary File 3. 

**Fig. 2:**
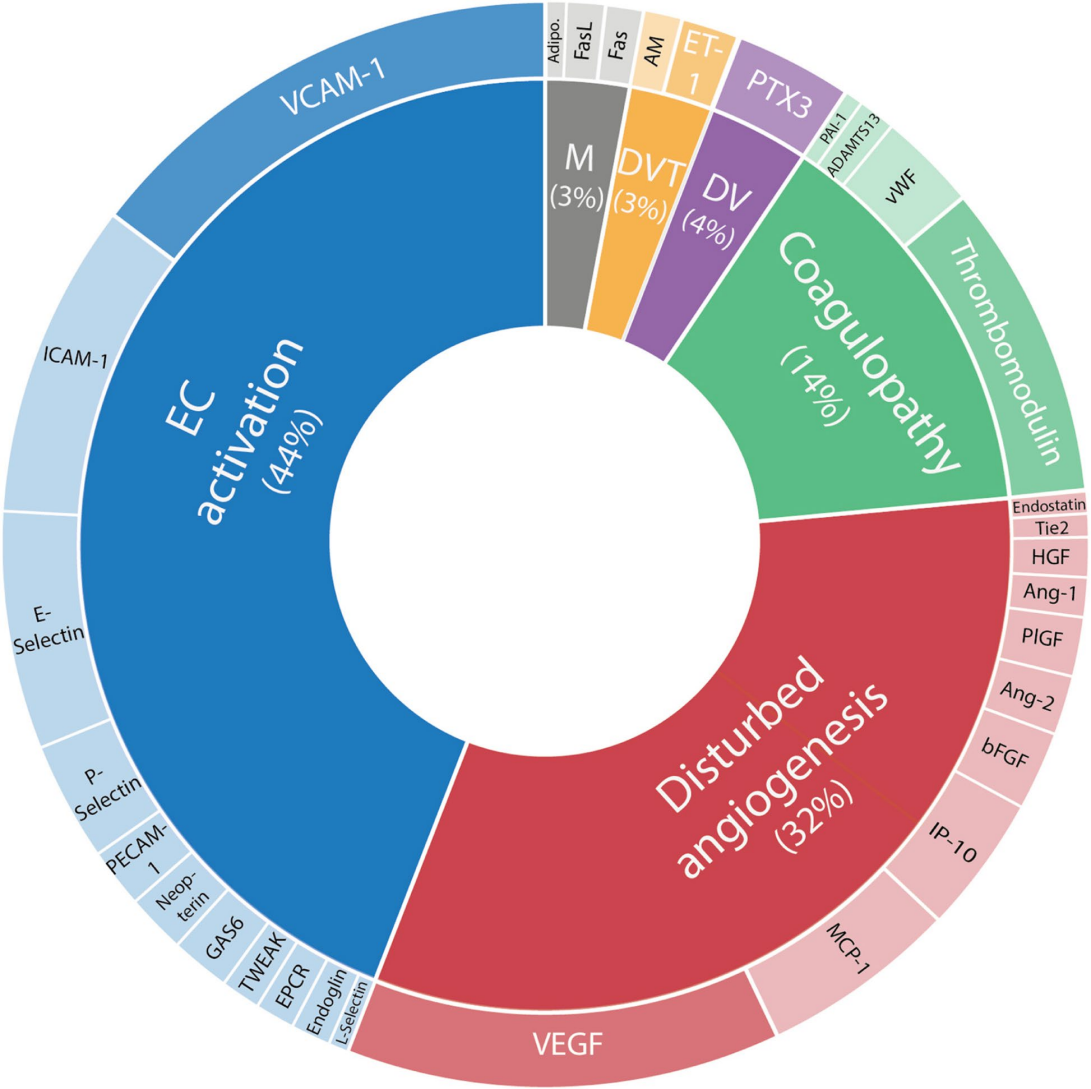
Ferris Wheel Plot. Ferris Wheel Plot summarizing the identified EC markers in SLE per category of dominant EC dysfunction of each EC marker. The surface area of each EC marker represents the number of articles with EC marker data on the corresponding dysfunction. The EC marker with the highest number of articles in which the EC marker was reported as dysregulated, can be recognized by the darkened background color and white text). EC = endothelial cell, DV = disturbed vasculogenesis, DVT = defective vascular tone control, M = mixed (adipocytokines and EC apoptosis). Markers in Ferris Wheel Plot: ADAMTS13: A Disintegrin-like and Metalloprotease with Thrombospondin Type 1 Motif Adipo: Adiponectin AM: Adrenomedullin Ang-1: Angiopoietin-1 Ang-2: Angiopoietin-2 bFGF: basic fibroblast growth factor Endoglin Endostatin EPCR: Endothelial protein C receptor E-Selectin ET-1: Endothelin-1 FasL: Fas ligand GAS6: growth arrest-specific gene 6 HGF: Hepatocyte growth factor ICAM-1: Intercellular Adhesion Molecule 1 IP-10: interferon-inducible protein 10 L-Selectin MCP-1: Monocyte chemoattractant protein-1 Neopterin PAI-1: Plasminogen activator inhibitor-1 PECAM-1: Platelet and endothelial cell adhesion molecule 1 PlGF: Placental growth factor P-Selectin PTX3: Pentraxin-3 sEPCR: Soluble endothelial protein C receptor Tie2: Tyrosine kinase with immunoglobulin-like and EGF-like domain 2 TM: Thrombomodulin VCAM-1: Vascular Cell Adhesion Molecule 1 VEGF: Vascular Endothelial Growth-Factor VWF: Von Willebrand Factor TWEAK: Tumor necrosis factor (TNF)-like weak inducer of apoptosis

SLE-related EC markers reflecting different mechanisms of EC function were identified from the selected articles. Next, these markers were clustered according to their predominant endothelial (dys)function. A classification system adapted from Mostmans et al. was used [34]. Vasculogenesis and angiogenesis are separately described in this system, with resp. involvement of recruitment, mobilization and in situ differentiation of endothelial progenitor cells (EPC) versus proliferation and migration of mature ECs. The distribution of EC marker citations across the different endothelial dysfunction categories are visualized in Fig. 2. Although the clustering of EC marker citations per function illustrates a predominance of the ‘endothelial cell activation’ category (44%), the majority of the marker citations fall into four other endothelial dysfunction categories: ‘disturbed angiogenesis’ (32%), ‘coagulation cascade and complement impairment’ (14%), ‘disturbed vasculogenesis’ (4%) and ‘defective vascular tone control’ (3%). VCAM-1 was the most reported EC marker (*n* = 25 articles, ‘EC activation’) in the selected articles of this systematic review, followed by vascular endothelial growth factor (VEGF, *n* = 22, ‘disturbed angiogenesis’), ICAM-1 (*n* = 16, ‘EC activation’) and thrombomodulin (TM, *n* = 15, ‘coagulation cascade and complement impairment’).

**Fig. 3:**
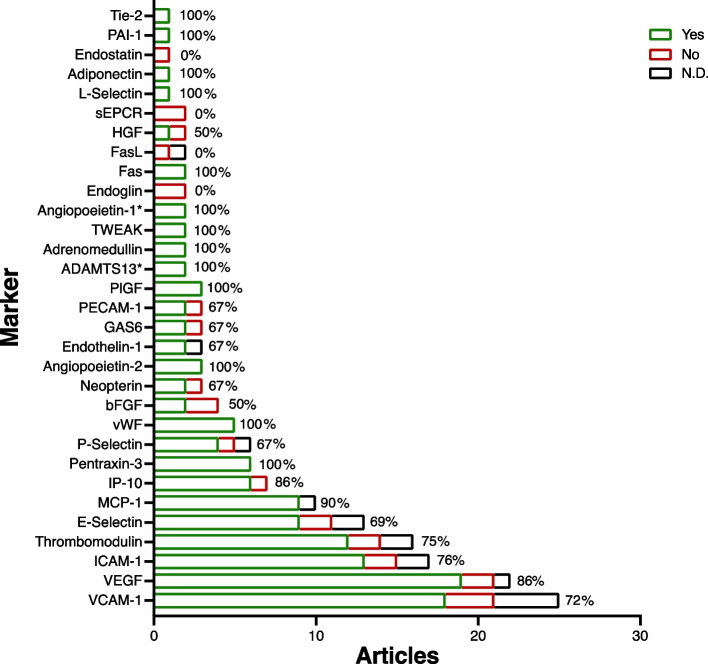
Overview of the number of articles per SLE-related EC marker. Overview of the number of articles per EC marker, indicating for each marker on the y-axis whether there was a significant increase (no symbol) or decrease (*) of plasma/serum levels when compared to healthy controls (yes (green box), no (red box), not determined (N.D.) (black box). The percentage indicates the number of articles per EC marker that showed a significant increase of EC marker levels (% ‘yes’ of total citations). EC- Endothelial cell

For each EC marker, the number of articles that reported significantly altered plasma/serum levels for SLE patients versus healthy controls was determined (Fig. 3).

Next, the reported correlations between the plasma/serum levels of each EC marker and validated SLE disease activity indexes were assessed. This figure can be found in Supplementary file 2, figure A.

**Fig. 4:**
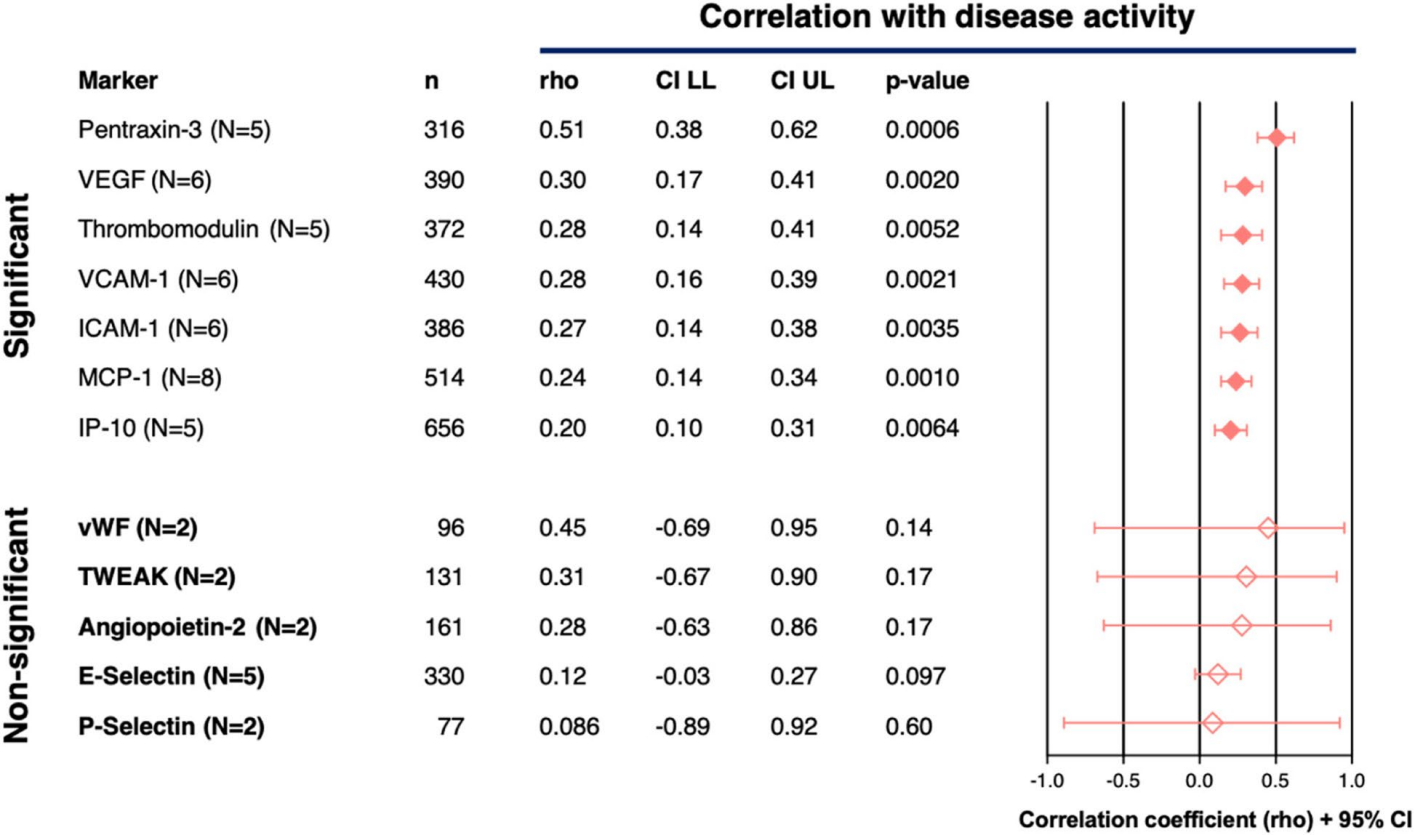
Meta-analyses (Spearman’s rho correlations). Meta-analysis of 12/31 identified EC markers fulfilling the inclusion criteria for meta-analysis. Overview of overall Spearman’s correlations (rho, with 95% CI) between SLE disease activity and serum/plasma levels of each included EC marker. *n* = total number of SLE patients from all articles per EC marker. In the Forest Plot, significant correlations (95% CI does not include ‘0’ and *p* < 0.05) are indicated by black text and filled diamonds (♦) and non-significant correlations (95% CI includes ‘0’ and *p* > 0.05) are indicated by grey text and open diamonds (♢). Calculated *p*-values are based on Fisher's Z Transformation. Markers that are not produced or expressed solely/primarily by EC: Pentraxin-3, GAS6, MCP-1, P-Selectin, TWEAK and IP-1

**Fig. 5:**
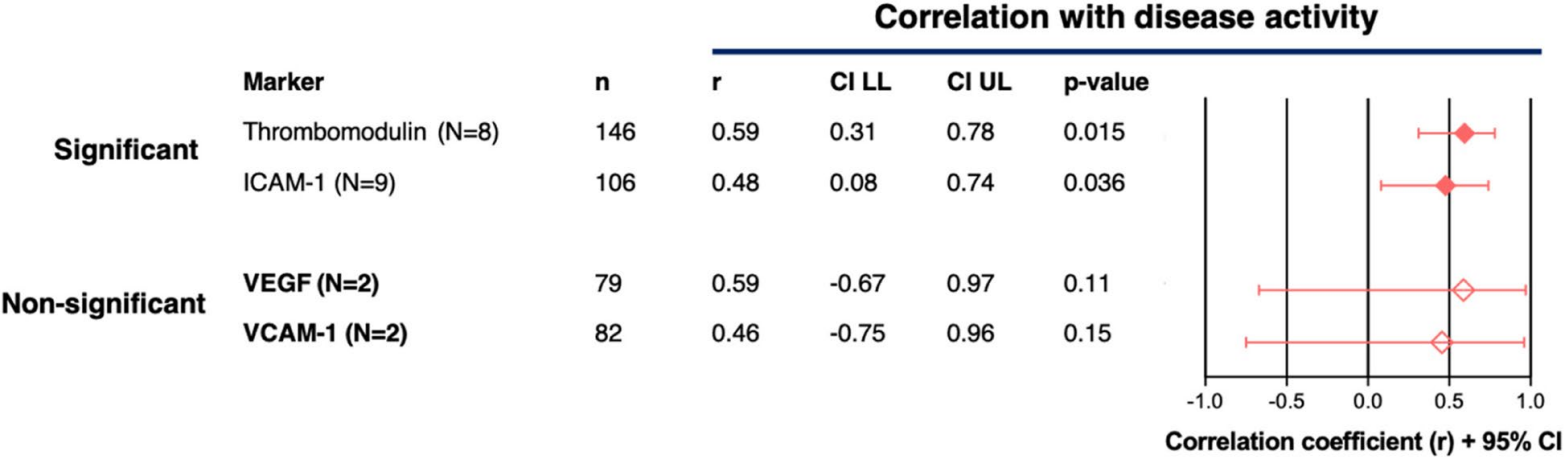
Meta-analyses Pearson’s r correlations. Meta-analysis of 4/31 identified EC markers fulfilling the inclusion criteria for meta-analysis. Overview of overall Pearson’s correlations (r, with 95% CI) between SLE disease activity and serum/plasma levels of each included EC marker. *n* = total number of SLE patients from all articles per EC marker. In the Forest Plot, significant correlations (95% CI does not include ‘0’ and *p* < 0.05) are indicated by black text and filled diamonds (♦) and non-significant correlations (95% CI includes ‘0’ and *p* > 0.05) are indicated by grey text and open diamonds (♢) Calculated *p*-values are based on Fisher's Z Transformation

Twelve of the 31 identified EC markers fulfilled the inclusion criteria for performing a meta-analysis. Meta-analyses using Spearman’s rho correlation coefficients could be performed for 12/12 markers. Meta-analysis using Pearson’s r correlation coefficients could be performed for 4/12 markers. Meta-analysis of 7/12 markers (58.3%) showed significant Spearman’s correlations with disease activity (Fig. 4, EC markers not bold). These seven markers were: Pentraxin-3 (‘disturbed vasculogenesis’), VCAM-1 and ICAM-1 (both EC Activation), Thrombomodulin (‘coagulopathy’), MCP-1, IP-10 and VEGF (all ‘disturbed angiogenesis’). The other five EC markers were dysregulated in SLE patients when compared to healthy controls (Fig. 3), but meta-analysis did not identify significant correlations with disease activity (Fig. 4, EC markers in bold). These five dysregulated EC markers without associations with disease activity were: von Willebrand factor (vWF, ‘coagulation and complement cascade’), angiopoietin-2 (Ang-2), (‘disturbed angiogenesis’), P-selectin, TWEAK, and E-selectin (all EC activation). In meta-analysis with Pearon’s correlations, Thrombomodulin and ICAM-1 showed significant correlations (Fig. 5, markers not bold). VCAM-1 and VEGF were dysregulated without correlation with disease activity Fig. 5, markers bold). The extracted results and statistical methods of the papers selected for disease activity correlation meta-analysis are summarized in Supplementary File 4. In Supplementary File 5 (Figures S5A-S5P), details of the meta-analyses for each marker are provided. There was no evidence of significant publication bias either with Begg's test or Egger's test, if applicable. Publication bias was not assessed if there were inadequate numbers of included studies to properly assess a funnel plot or more advanced regression-based assessments.

## Discussion

This systematic literature review provides the first comprehensive literature overview of EC markers that are dysregulated in SLE, as indicated primarily from cross-sectional studies. From 123 selected articles, 31 EC markers with dysregulation in SLE were identified. We found five EC markers to be dysregulated in SLE, but without correlations with disease activity; Angiopoeitin-2, vWF, P-Selectin, TWEAK and E-Selectin. This could mean that the endothelium stays in an active and dysregulated state in SLE, even in case of low disease activity or disease remission. In addition, for seven EC markers, meta-analysis demonstrated significant correlations between serum/plasma levels and SLE disease activity. Consequently, these EC-associated proteins may represent (novel) biomarkers for monitoring disease activity in SLE and/or identifying patients at risk of premature atherosclerosis.

The diversity in EC dysfunction categories illustrates the multifaceted nature of SLE-associated vascular disease. VCAM-1 and ICAM-1 are both adhesion molecules involved in the endothelial interaction with inflammatory cells, including lymphocytes, further promoting EC activation. In addition, expression of these adhesion molecules on EC is important in the attraction of immune cells to sites of inflammation (i.e. skin, kidney, joint, etc.), which is also an important aspect in the development of atherosclerosis and plaque growth [35]. Despite the similarities between the two adhesion molecules, VCAM-1 expression is largely restricted to lesions and lesion-predisposed regions, whereas ICAM-1 expression extends into uninvolved lesion-protected regions. VEGF, a ligand of the VEGF receptors 1 and 2, is secreted by ECs and functions as a key mediator of proliferation and migration of ECs. Moreover, VEGF is considered to be an important regulator of angiogenesis type of neovascularization, both in normal and pathological conditions [36]. VEGF-induced formation of microvessels in atherosclerotic lesions contributes to plaque instability and rupture causing cardiovascular events [13, 37]. Of note, elevated VEGF levels have not only been reported in the serum of SLE patients, but also in RA and myositis patients who are known to have an increased CVD risk [38]. Thrombomodulin (TM) is a thrombin receptor that is expressed on the vascular EC surface. TM binds thrombin and thereby inhibits the procoagulant actions of this ligand via the protease-activated receptor 1 (PAR-1). In case of EC injury, TM sheds from the membrane and is detectable in plasma and serum in its soluble form [39]. Therefore, soluble TM levels are considered to reflect EC damage.

Our meta-analysis demonstrated a positive correlation between some EC marker levels and disease activity for the majority of the highly cited markers (i.e. ICAM-1, VEGF and TM and VCAM-1). However, it is of important note that these correlations are weak (Spearman’s rho values between 0.21–0.40), which could mean that these markers also reflect endothelial dysregulation, even in case of low disease activity. Meta-analysis revealed a weak positive correlation with disease activity for MCP-1 (*ρ* = 0.24) (‘disturbed angiogenesis’) and a moderate correlation with pentraxin-3 (*ρ* = 0.51, ‘disturbed vasculogenesis’). For GAS6, it was not possible to perform meta-analysis since there were not enough studies included with the same reported correlation coefficient. However, GAS6 deserves additional focus as being recently implicated as a potential biomarker in the context of SLE [30, 40]. GAS6, a vitamin K-dependent growth factor, is expressed in different cell types, including ECs, mesangial cells and macrophages [41]. GAS6 is a ligand of the receptor tyrosine kinase Axl and its blood levels are elevated in inflammatory conditions, such as sepsis and SLE [42]. Several studies have implicated GAS6 in (premature) atherosclerosis [41]. By promoting smooth muscle cell survival, migration and accumulation within the atherosclerotic plaque as well as vascular remodelling, GAS6 generates a more stable plaque with a strengthened fibrous cap [40, 43]. In a Korean SLE study GAS6, serum levels not only correlated with SLEDAI (*r* = 0.51, *p* < 0.001), but also with a change in SLEDAI over time (*r* = 0.52, *p* < 0.001) [44]. Another example of a novel marker in the context of SLE is pentraxin-3, with its first citation dating from 2014 [45]. Pentraxin-3 is an acute-phase inflammation protein produced by several cell types, involved in EC dysfunction and atherosclerosis through various mechanisms [46]. For instance, pentraxin-3 decreases the synthesis of nitric oxide (NO), contributing to a defective vascular tone control [47]. In addition, pentraxin-3 affects the lipid metabolism in human macrophages by stimulating the uptake of oxLDL and inhibiting cholesterol efflux [48], thereby contributing to the initial stages of EC dysfunction and consequent atherosclerosis. Similar to GAS6, a longitudinal study showed that the pentraxin-3 levels correlated well with the changes in SLEDAI over time [49]. Hence, GAS6 and pentraxin-3 appear to accurately reflect SLE disease activity changes and could therefore be useful biomarkers to monitor ongoing CVD risk and efficiency of (novel) treatments.

Our meta-analysis revealed non-significant correlations with disease activity for the following markers: von Willebrand factor (vWF), angiopoietin-2 (Ang-2), P-selectin, TWEAK, and E-selectin. This implies that these EC markers are elevated in SLE patients, irrespective of disease activity. This could be a random effect but could also mean that SLE patients in remission or low disease activity might still be at ongoing risk for widespread EC dysfunction. This would suggest that these specific EC-associated proteins have added value as (bio)markers for predicting the ongoing risk for EC dysfunction and consequent premature atherosclerosis in all SLE patients, including those with low disease activity or disease remission. Longitudinal studies with sampling over time in different disease states per patient will need to be performed to shed more light on this issue.

The two most frequently measured markers in studies with a longitudinal design were VCAM-1 and IP-10. In these longitudinal studies, changes in disease activity over time were accurately reflected by VCAM-1 and IP-10 levels [39, 50–53]. For both of these markers all but one of these studies (resp. *n* = 8 and *n* = 5), reported significant correlation coefficients. Hence, VCAM-1 and IP-10 may presumably reflect progressive disease activity over time rather than predicting ongoing risk for EC dysfunction in patients in remission.

For ADAMTS13 (‘coagulation cascade and complement impairment’), a vWF-cleaving protease, significant negative correlations with disease activity were consistently reported [33, 54, 55]. However, a lack of studies with suitable correlation coefficients prevented meta-analysis for ADAMTS13. Similarly, despite multiple studies with significant correlations for adrenomedullin (AM) and endothelin-1 (ET-1) (both ‘defective vascular tone control’) with disease activity, a meta-analysis could not be performed for these markers. Al-Yasaky and colleagues showed a high correlation between AM levels and SLEDAI (*r* = 0.62, *p* < 0.001) [56]. Despite a significant disease activity correlation coefficient in a second study, the use of linear regression prevented a meta-analysis in our study for this marker [57]. For ET-1, correlation coefficients (r values) were reported for only one out of multiple studies with significant correlations [58–60], thereby also limiting to perform a meta-analysis. Although these studies were not eligible for inclusion in the meta-analysis, the results of the performed studies do suggest that these EC markers play a crucial role in the EC dysfunction in SLE and thus in the development of atherosclerosis.

Our study has some limitations. As mentioned above, some of the selected articles did not report (compatible) correlation coefficients, especially when a non-significant correlation was observed. Those studies could not be included in the meta-analysis, which might have led to skewing of the meta-analysis outcome. This could have resulted in less or even no significant (positive) correlations between disease activity and these markers. Some EC-derived proteins have been demonstrated only recently to be dysregulated in SLE. If these ‘novel’ markers were not reported in at least two articles and tested for correlation with disease activity using Spearman’s or Pearson’s rank test, they could not be used for meta-analysis. For some markers, only a few studies were included in meta-analysis. These small studies (i.e. the separate EC markers) are more likely to be affected by publication and selection biases in meta-analysis. An important aspect is the fact that co-variables such as disease duration, specific medication use, BMI and blood pressure could not be further investigated by meta-regression analysis. These data could not be (fully) retrieved from the studies, or available information was not complete. For instance, use of some medications was sometimes mentioned but duration or cumulative use was not taken into account. Disease duration from the studies in our meta-analysis (*n* = 41) differed by type of documentation and this variable had some lack of data (of which *n* = 16 studies with unknown data). Minimum mentioned disease duration was 18 months and the maximum was 19 years. Range of age of the included patients was 8 to 77 years but differed in mentioning by mean or median. Therefore, we did not perform a meta-regression analysis with these factors.

Since we fully focused on ECs, our study has the limitation of only including those studies describing proteins directly produced by ECs or activating these cells. Moreover, some EC markers are not *only* ‘endothelial cell’-specific. A few of the aforementioned EC markers in this review are also produced and/or secreted by other cell types (e.g. fibroblasts, macrophages, dendritic cells or neutrophils).

## Conclusion

This study provides some clarity in the eminent complex field of EC markers as biomarkers for SLE. The identified EC-associated proteins in SLE cover a wide range of different EC functions. Intriguingly, SLE-induced EC marker dysregulation was also seen irrespective of disease activity. This systematic literature review and meta-analysis highlights several well-studied, but also some relatively novel EC-associated proteins that could serve as biomarkers for developing premature atherosclerosis. More insights in these EC markers and their predictive value for premature atherosclerosis, and therefore risk for cardiovascular damage, may be obtained from future studies with a longitudinal design. Given the low number of paediatric patients in the reported studies, but the concomitantly higher incidence of CVD and morbidity rates at young adult age in this patient group, there is especially an urge for studies in paediatric SLE patients.

## Supplementary Information

**Additional file 1.** Flowchart of search and selection process of the articles.

**Additional file 2.** Supplementary File 2 provides an overview of all included studies in chronological order and their characteristicscorrelation with disease activity, method of blood sample analysis. Figure A. Overview of the number of articles per EC marker reporting whether or not a significant correlation between plasma/serum levels of each EC marker and validated SLE disease activity index. 

**Additional file 3.** Quality assessment overview.

**Additional file 4.** Table showing the statistical methods and reported correlations of individual markers selected for meta-analysis, as well as the *P*-value calculations for overall correlation coefficients.

**Additional file 5.** Meta-analyses per marker. Figures S5A-S5L. Spearman’s rho correlations. Figures S5M-S5P. Pearson’s r correlations.

## Data Availability

The datasets used and/or analysed during the current study are available from the corresponding author on reasonable request.

## Abbreviations

ACR: American College of Rheumatology
ADAMTS13: A Disintegrin-like and Metalloprotease with Thrombospondin Type 1 Motif
AM: Adrenomedullin
Ang-1: Angiopoietin-1
Ang-2: Angiopoietin-2
anti-dsDNA: Anti-double stranded DNA
bFGF: Basic fibroblast growth factor
BILAG: British Isles Lupus Assessment Group
CVD: Cardiovascular diseases
cSLE: Childhood-onset Systemic Lupus Erythematosus
ECLAM: European Consensus Lupus Activity Measurement
EC: Endothelial cell
EPC: Endothelial progenitor cell
EPCR: Endothelial protein C receptor
E-Selectin: Endothelial Selectin
ET-1: Endothelin-1
FasL: Fas ligand
GAS6: Growth arrest-specific gene 6
HC: Healthy Controls
HGF: Hepatocyte growth factor
ICAM-1: Intercellular Adhesion Molecule 1
IP-10: Interferon-inducible protein 10
L-Selectin: Leukocyte Selectin
MCP-1: Monocyte chemoattractant protein-1
PAI-1: Plasminogen activator inhibitor-1
PECAM-1: Platelet and endothelial cell adhesion molecule 1
PlGF: Placental growth factor
P-Selectin: Platelet Selectin
PTX3: Pentraxin-3
sEPCR: Soluble endothelial protein C receptor
SLAM(-R): Systemic Lupus Activity Measure(-Revised)
SLEDAI: Systemic Lupus Erythematosus Disease Activity Index
SLICC: Systemic Lupus International Collaborating Clinics
LN: Lupus Nephritis
Tie2: Tyrosine kinase with immunoglobulin-like and EGF-like domain 2
TM: Thrombomodulin
VCAM-1: Vascular Cell Adhesion Molecule 1
VEGF: Vascular Endothelial Growth-FActor
VWF: Von Willebrand Factor
TWEAK: Tumor necrosis factor (TNF)-like weak inducer of apoptosis

## References

[1] Chen HJ, Tas SW, de Winther MPJ (2020). Type-I interferons in atherosclerosis. J Exp Med..

[2] Maracle CX, Agca R, Helder B, Meeuwsen JAL, Niessen HWM, Biessen EAL (2018). Noncanonical NF-κB signaling in microvessels of atherosclerotic lesions is associated with inflammation, atheromatous plaque morphology and myocardial infarction. Atherosclerosis.

[3] Vavlukis M, Pop-Gjorcevab D, Poposka L, Sandevska E, Kedev S (2021). Myocardial Infarction in Systemic Lupus Erythematosus - the Sex-Specific Risk Profile. Curr Pharm Des..

[4] Restivo V, Candiloro S, Daidone M, Norrito R, Cataldi M, Minutolo G (2021). Systematic review and meta-analysis of cardiovascular risk in rheumatological disease: symptomatic and non-symptomatic events in rheumatoid arthritis and systemic lupus erythematosus. Autoimmun Rev..

[5] Levy DM, Kamphuis S (2012). Systemic lupus erythematosus in children and adolescents. Pediatr Clin North Am.

[6] Tydén H, Lood C, Gullstrand B, Jönsen A, Nived O, Sturfelt G (2013). Increased serum levels of S100A8/A9 and S100A12 are associated with cardiovascular disease in patients with inactive systemic lupus erythematosus. Rheumatology (Oxford).

[7] Manzi S, Meilahn EN, Rairie JE, Conte CG, Medsger TA, Jansen-McWilliams L (1997). Age-specific incidence rates of myocardial infarction and angina in women with systemic lupus erythematosus: comparison with the Framingham Study. Am J Epidemiol.

[8] Conrad N, Verbeke G, Molenberghs G, Goetschalckx L, Callender T, Cambridge G (2022). Autoimmune diseases and cardiovascular risk: a population-based study on 19 autoimmune diseases and 12 cardiovascular diseases in 22 million individuals in the UK. Lancet.

[9] Boodhoo KD, Liu S, Zuo X (2016). Impact of sex disparities on the clinical manifestations in patients with systemic lupus erythematosus: A systematic review and meta-analysis. Medicine (Baltimore).

[10] Vogel B, Acevedo M, Appelman Y, Bairey Merz CN, Chieffo A, Figtree GA (2021). The Lancet women and cardiovascular disease Commission: reducing the global burden by 2030. Lancet.

[11] Groot N, Shaikhani D, Teng YKO, de Leeuw K, Bijl M, Dolhain R (2019). Long-term clinical outcomes in a cohort of adults with childhood-onset systemic lupus erythematosus. Arthritis Rheumatol.

[12] Westerweel PE, Luyten RK, Koomans HA, Derksen RH, Verhaar MC (2007). Premature atherosclerotic cardiovascular disease in systemic lupus erythematosus. Arthritis Rheum.

[13] Al-Soudi A, Kaaij MH, Tas SW (2017). Endothelial cells: From innocent bystanders to active participants in immune responses. Autoimmun Rev.

[14] Perez-Sanchez C, Ruiz-Limon P, Aguirre MA, Bertolaccini ML, Khamashta MA, Rodriguez-Ariza A (2012). Mitochondrial dysfunction in antiphospholipid syndrome: implications in the pathogenesis of the disease and effects of coenzyme Q(10) treatment. Blood.

[15] Mak A, Kow NY, Schwarz H, Gong L, Tay SH, Ling LH (2017). Endothelial dysfunction in systemic lupus erythematosus - a case-control study and an updated meta-analysis and meta-regression. Sci Rep.

[16] Libby P (2021). The changing landscape of atherosclerosis. Nature.

[17] Theodorou K, Boon RA (2018). Endothelial cell metabolism in atherosclerosis. Front Cell Dev Biol.

[18] Teixeira V, Tam LS (2017). Novel Insights in systemic lupus erythematosus and atherosclerosis. Front Med (Lausanne).

[19] Skaggs BJ, Hahn BH, McMahon M (2012). Accelerated atherosclerosis in patients with SLE–mechanisms and management. Nat Rev Rheumatol.

[20] Ritis K, Doumas M, Mastellos D, Micheli A, Giaglis S, Magotti P (2006). A novel C5a receptor-tissue factor cross-talk in neutrophils links innate immunity to coagulation pathways. J Immunol.

[21] Schonenberg-Meinema D, Bergkamp SC, Nassar-Sheikh Rashid A, van der Aa LB, de Bree GJ, Ten Cate R, Cutolo M, Hak AE, Hissink Muller PC, van Onna M, Kuijpers TW, Smith V, van den Berg JM (2021). Nailfold capillary abnormalities in childhood-onset systemic lupus erythematosus: a cross-sectional study compared with healthy controls. Lupus..

[22] Bergkamp SC, Schonenberg-Meinema D, Nassar-Sheikh Rashid A, Melsens K, Vanhaecke A, Boumans MJH (2021). Reliable detection of subtypes of nailfold capillary haemorrhages in childhood-onset systemic lupus erythematosus. Clin Exp Rheumatol.

[23] Shamseer L, Moher D, Clarke M, Ghersi D, Liberati A, Petticrew M (2015). Preferred reporting items for systematic review and meta-analysis protocols (PRISMA-P) 2015: elaboration and explanation. BMJ.

[24] NIH QA. National Institutes of Health (2014). Quality Assessment Tool for Observational Cohort and Cross-Sectional Studies. Available online at: https://www.nhlbi.nih.gov/health-pro/guidelines/in-develop/cardiovascular-risk-reduction/tools/cohort. (Accessed Dec 2020). 2020.

[25] Stock W (1987). Statistical Methods for Meta-Analysi's:Larry V. Hedges and Ingram Olkin Orlando FL: Academic Press, 1985, 369 pp., approx. $49.00. Appl Psychol Meas.

[26] Suurmond R, van Rhee H, Hak T (2017). Introduction, comparison, and validation of meta-essentials: a free and simple tool for meta-analysis. Res Synth Methods.

[27] Sánchez-Meca J, Marín-Martínez F (2008). Confidence intervals for the overall effect size in random-effects meta-analysis. Psychol Methods.

[28] el-Gamal YM, Heshmat NM, el-Kerdany TH, Fawzy AF (2004). Serum thrombomodulin in systemic lupus erythematosus and juvenile idiopathic arthritis. Pediatr Allergy Immunol.

[29] Heshmat NM, El-Kerdany TH (2007). Serum levels of vascular endothelial growth factor in children and adolescents with systemic lupus erythematosus. Pediatr Allergy Immunol.

[30] Elhelaly N, Elhawary I, Alaziz I, Alsalam M, Elfishawy H, Sherif M (2009). The clinical utility of vascular endothelial growth factor as predictive marker for systemic lupus erythematosus activity in children and adolescents. J Biol Sci..

[31] Sahin S, Adrovic A, Barut K, Durmus S, Gelisgen R, Uzun H (2017). Pentraxin-3 levels are associated with vasculitis and disease activity in childhood-onset systemic lupus erythematosus. Lupus.

[32] Zhang CX, Cai L, Shao K, Wu J, Zhou W, Cao LF (2018). Serum IP-10 is useful for identifying renal and overall disease activity in pediatric systemic lupus erythematosus. Pediatr Nephrol.

[33] Lee WF, Wu CY, Yang HY, Lee WI, Chen LC, Ou LS (2019). Biomarkers associating endothelial Dysregulation in pediatric-onset systemic lupus erythematous. Pediatr Rheumatol Online J.

[34] Mostmans Y, Cutolo M, Giddelo C, Decuman S, Melsens K, Declercq H (2017). The role of endothelial cells in the vasculopathy of systemic sclerosis: a systematic review. Autoimmun Rev.

[35] Cook-Mills JM, Marchese ME, Abdala-Valencia H (2011). Vascular cell adhesion molecule-1 expression and signaling during disease: regulation by reactive oxygen species and antioxidants. Antioxid Redox Signal.

[36] Jośko J, Gwóźdź B, Jedrzejowska-Szypułka H, Hendryk S (2000). Vascular endothelial growth factor (VEGF) and its effect on angiogenesis. Med Sci Monit.

[37] Camaré C, Pucelle M, Nègre-Salvayre A, Salvayre R (2017). Angiogenesis in the atherosclerotic plaque. Redox Biol.

[38] Kikuchi K, Kubo M, Kadono T, Yazawa N, Ihn H, Tamaki K (1998). Serum concentrations of vascular endothelial growth factor in collagen diseases. Br J Dermatol.

[39] Boehme MW, Raeth U, Galle PR, Stremmel W, Scherbaum WA (2000). Serum thrombomodulin-a reliable marker of disease activity in systemic lupus erythematosus (SLE): advantage over established serological parameters to indicate disease activity. Clin Exp Immunol.

[40] Nakano T, Kawamoto K, Higashino K-i, Arita H (1996). Prevention of growth arrest-induced cell death of vascular smooth muscle cells by a product of growth arrest-specific gene, gas6. FEBS Lett.

[41] Laurance S, Lemarié CA, Blostein MD (2012). Growth arrest-specific gene 6 (gas6) and vascular hemostasis. Adv Nutr.

[42] Wu Q, Guan SY, Dan YL, Zhao CN, Mao YM, Liu LN (2019). Circulating pentraxin-3 levels in patients with systemic lupus erythematosus: a meta-analysis. Biomark Med.

[43] Fridell YW, Villa J, Attar EC, Liu ET (1998). GAS6 induces Axl-mediated chemotaxis of vascular smooth muscle cells. J Biol Chem.

[44] Kim HA, Nam JY, Jeon JY, An JM, Jung JY, Bae CB (2013). Serum growth arrest-specific protein 6 levels are a reliable biomarker of disease activity in systemic lupus erythematosus. J Clin Immunol.

[45] Shimada Y, Asanuma YF, Yokota K, Yoshida Y, Kajiyama H, Sato K (2014). Pentraxin 3 is associated with disease activity but not atherosclerosis in patients with systemic lupus erythematosus. Mod Rheumatol.

[46] Carrizzo A, Procaccini C, Lenzi P, Fusco C, Villa F, Migliarino S (2019). PTX3: an inflammatory protein modulating ultrastructure and bioenergetics of human endothelial cells. Immun Ageing.

[47] Zlibut A, Bocsan IC, Agoston-Coldea L (2019). Chapter Five - Pentraxin-3 and endothelial dysfunction. Adv Clini Chem..

[48] Liu W, Jiang J, Yan D, Li D, Li W, Ma Y (2014). Pentraxin 3 promotes oxLDL uptake and inhibits cholesterol efflux from macrophage-derived foam cells. Exp Mol Pathol.

[49] Assandri R, Monari M, Colombo A, Dossi A, Montanelli A (2015). Pentraxin 3 plasma levels and disease activity in systemic lupus erythematosus. Autoimmune Dis.

[50] Horák P, Scudla V, Hermanovó Z, Pospisil Z, Faltýnek L, Budiková M (2001). Clinical utility of selected disease activity markers in patients with systemic lupus erythematosus. Clin Rheumatol.

[51] de Leeuw K, Smit AJ, de Groot E, van Roon AM, Kallenberg CG, Bijl M (2009). Longitudinal study on premature atherosclerosis in patients with systemic lupus erythematosus. Atherosclerosis.

[52] Lewis MJ, Vyse S, Shields AM, Zou L, Khamashta M, Gordon PA (2016). Improved monitoring of clinical response in Systemic Lupus Erythematosus by longitudinal trend in soluble vascular cell adhesion molecule-1. Arthritis Res Ther.

[53] Kong KO, Tan AW, Thong BY, Lian TY, Cheng YK, Teh CL (2009). Enhanced expression of interferon-inducible protein-10 correlates with disease activity and clinical manifestations in systemic lupus erythematosus. Clin Exp Immunol.

[54] Martin-Rodriguez S, Reverter JC, Tàssies D, Espinosa G, Heras M, Pino M (2015). Reduced ADAMTS13 activity is associated with thrombotic risk in systemic lupus erythematosus. Lupus.

[55] Muscal E, Edwards RM, Kearney DL, Hicks JM, Myones BL, Teruya J (2011). Thrombotic microangiopathic hemolytic anemia with reduction of ADAMTS13 activity: initial manifestation of childhood-onset systemic lupus erythematosus. Am J Clin Pathol.

[56] Al-Yasaky, A. Z. M. M. a. N. Z. Hala Mahfouz*Soluble thrombomoduline (STM) and human adrenomedullin (AM) in systemic lupus erythematosus and their relation to disese activity and renal affection . Egypt Rheumatol Rehab. 2005;32(2):217-33.

[57] Mak A, Cheung BM, Mok CC, Leung R, Lau CS (2006). Adrenomedullin–a potential disease activity marker and suppressor of nephritis activity in systemic lupus erythematosus. Rheumatology (Oxford).

[58] Kuryliszyn-Moskal A, Ciolkiewicz M, Klimiuk PA, Sierakowski S (2009). Clinical significance of nailfold capillaroscopy in systemic lupus erythematosus: correlation with endothelial cell activation markers and disease activity. Scand J Rheumatol.

[59] Ciolkiewicz M, Kuryliszyn-Moskal A, Klimiuk PA (2010). Analysis of correlations between selected endothelial cell activation markers, disease activity, and nailfold capillaroscopy microvascular changes in systemic lupus erythematosus patients. Clin Rheumatol.

[60] Hajialilo M, Tayari P, Ghorbanihaghjo A, Khabbazi A, Malek Mahdavi A, Rashtchizadeh N (2018). Relationship between serum vascular cell adhesion molecule-1 and endothelin-1 levels with organ involvement and disease activity in systemic lupus erythematosus patients. Lupus.

