# Supplementary material for: Dysregulated endothelial cell markers in systemic lupus erythematosus: a systematic review and meta-analysis
Source: J Inflamm (Lond). 2023 May 16;20:18. doi: 10.1186/s12950-023-00342-1 (PMC10189957; doi:10.1186/s12950-023-00342-1)
Supplement: Supplementary file 1 — Additional file 1. Flowchart of search and selection process of the articles. [file 12950_2023_342_MOESM1_ESM.docx]

**Supplementary File 1**Flowchart of search and selection process of the articles

*Search was performed initially in July 2020. Search has been updated in April, 2022.*

Search terms per database:

**Embase.com**

('endothelium cell'/exp OR 'endothelial progenitor cell'/de OR 'biological marker'/de/mj OR 'marker'/de/mj OR (endothel* OR vascul*):ab,ti,kw OR (marker* OR biomarker*):ti) **AND** ('systemic lupus erythematosus'/exp/mj OR 'SLEDAI'/de OR (SLE OR SLEDAI OR lupus*):ti) **AND** ('pathogenesis'/de OR 'disease activity'/de OR 'SLEDAI'/de OR 'physician global assessment'/de OR 'disease marker'/de OR (pathogenes* OR SLEDAI* OR Systemic-Lupus-Erythematosus-Disease-Activity-Index* OR (disease* NEAR/3 activit*) OR BILAG* OR british-isles-lupus-assessment-group* OR PGA OR physician-global-assessment*):ab,ti,kw) *AND [english]/lim* *NOT ((animal/exp OR animal*:de OR nonhuman/de) NOT ('human'/exp)) NOT ([Conference Abstract]/lim OR [Editorial]/lim OR [Letter]/lim OR [Note]/lim)*

**Medine (Ovid)**

(exp Endothelial Cells/ OR * Biomarkers/ OR (endothel* OR vascul*).ab,ti,kf. OR (marker* OR biomarker*).ti.) **AND** (exp * Lupus Erythematosus, Systemic/ OR (SLE OR SLEDAI OR lupus*).ti.) **AND** ((pathogenes* OR SLEDAI* OR Systemic-Lupus-Erythematosus-Disease-Activity-Index* OR (disease* ADJ3 activit*) OR BILAG* OR british-isles-lupus-assessment-group* OR PGA OR physician-global-assessment*).ab,ti,kf.) *AND english.lg.* *NOT (exp animals/ NOT humans/) NOT (letter* OR news OR comment* OR editorial* OR congres* OR abstract* OR book* OR chapter* OR dissertation abstract*).pt.*

**Web of Science**

(TS=(endothel* OR vascul*) OR TI=( marker* OR biomarker*)) **AND** (TI=(SLE OR SLEDAI OR lupus*)) **AND** (TS=(pathogenes* OR SLEDAI* OR Systemic-Lupus-Erythematosus-Disease-Activity-Index* OR (disease* NEAR/2 activit*) OR BILAG* OR british-isles-lupus-assessment-group* OR PGA OR physician-global-assessment*)) *AND LA=English AND DT=(Article OR Review)*

**Cochrane Central**

((endothel* OR vascul*):ab,ti,kw OR (marker* OR biomarker*):ti) **AND** ((SLE OR SLEDAI OR lupus*):ti) **AND** ((pathogenes* OR SLEDAI* OR Systemic-Lupus-Erythematosus-Disease-Activity-Index* OR (disease* NEAR/3 activit*) OR BILAG* OR british-isles-lupus-assessment-group* OR PGA OR physician-global-assessment*):ab,ti,kw)

**Google Scholar**

Endothelial|endothelium|vascular intitle:SLE|SLEDAI|lupus pathogenesis|SLEDAI|“Systemic Lupus Erythematosus Disease Activity Index”|“disease activity”|BILAG|“british isles lupus assessment group”|PGA|“physician global assessment”
