# Supplementary material for: Dysregulated endothelial cell markers in systemic lupus erythematosus: a systematic review and meta-analysis
Source: J Inflamm (Lond). 2023 May 16;20:18. doi: 10.1186/s12950-023-00342-1 (PMC10189957; doi:10.1186/s12950-023-00342-1)
Supplement: Supplementary file 2 — Additional file 2. Supplementary File 2 provides an overview of all included studies in chronological order and their characteristicscorrelation with disease activity, method of blood sample analysis. Figure A. Overview of the number of articles per EC marker reporting whether or not a significant correlation between plasma/serum levels of each EC marker and validated SLE disease activity index. [file 12950_2023_342_MOESM2_ESM.docx]

| **Author, year** | **Studied markers(s)** | **Study design** | **No. of patients** | **Age pts (years)*** | **Disease Duration** | **No. controls** | **Disease activity measurement (mean/median, ± SD, range)** | **Longitudinal follow up (no. of patients)** | **No. of samples per patient in case of longitudinal** | **Blood** | **Analysis method** |
| --- | --- | --- | --- | --- | --- | --- | --- | --- | --- | --- | --- |
|  |  |  |  |  |  |  |  |  |  |  |  |
| **Al-Maini et al.**  **2000**  ***Oman*** | Fas | Prospective observational study | 39 | 20 (13-27) | 3.5 years | 22 | SLEDAI  7.39 ± 6.26 [0 - 42] | No |  | Serum | ELISA |
| **Boehme et al. 2000**  ***Germany*** | Thrombomodulin | Prospective observational study | 30 | 34 (16-65) | unknown | - | SLAM  56 (SLAM score 0–5), 38 (SLAM score 6–10), and 30 (SLAM score > 10). | Yes (30) | 2 | Serum | ELISA |
|  | VCAM-1 |  |  |  |  |  |  |  |  |  |  |
|  | ICAM-1 |  |  |  |  |  |  |  |  |  |  |
|  | VEGF |  |  |  |  |  |  |  |  |  |  |
| **Egerer et al. 2000**  ***Germany*** | ICAM-1 | Case control | 33 | 33 (18-54) | unknown | 40 | SLEDAI  9 ± 5 (1 - 22) | No |  | Serum | ELISA |
|  | E-Selectin |  |  |  |  |  |  |  |  |  |  |
| **Font et al. 2000**  ***Spain*** | L-Selectin | Case control | 42 | 28 (11-64) | 8.8 years | 30 | SLEDAI  N.M. | No |  | Serum | ELISA |
| **Frijns et al. 2001**  ***The Netherlands*** | Thrombomodulin | Case control | 76 | 38 | 10.7 years | 25 | SLEDAI  4 (0–12) | No |  | Serum | ELISA |
|  | vWF |  |  |  |  |  |  |  |  |  |  |
|  | P-Selectin |  |  |  |  |  |  |  |  |  |  |
|  | VCAM-1 |  |  |  |  |  |  |  |  |  |  |
| **Horák et al. 2001**  ***Czech Republic*** | Thrombomodulin | Prospective observational study | 52 | 39 (20-73) | 4 years | - | ECLAM | Yes | 2 | Serum & plasma | ELISA |
|  | VCAM-1 |  |  |  |  |  |  |  |  |  |  |
|  | ICAM-1 |  |  |  |  |  |  |  |  |  |  |
|  | Neopterin |  |  |  |  |  |  |  |  |  |  |
|  | Fas Ligand |  |  |  |  |  |  |  |  |  |  |
| **Nagahama et al. 2001**  ***Japan*** | Thrombomodulin | Case control | 22 | 42 (39-45) | unknown | 20 | - | No |  | Serum | ELISA |
|  | VCAM-1 |  |  |  |  |  |  |  |  |  |  |
|  | PECAM-1 |  |  |  |  |  |  |  |  |  |  |
|  | P-Selectin |  |  |  |  |  |  |  |  |  |  |
|  | E-Selectin |  |  |  |  |  |  |  |  |  |  |
| **Navarro et al. 2002**  ***Mexico*** | VEGF | Case control | 28 | 36 (20.6-52.7) | unknown | 24 | - | No |  | Plasma | ELISA |
|  | VCAM-1 |  |  |  |  |  |  |  |  |  |  |
| **Robak et al. 2002**  ***Poland*** | VEGF | Case control | 52 | 41 (17-76) | 62 months | 20 | SLAM  N.M. | No |  | Serum | ELISA |
|  | Endostatin |  |  |  |  |  |  |  |  |  |  |
|  | bFGF |  |  |  |  |  |  |  |  |  |  |
| **Sari et al. 2002**  ***Turkey*** | ICAM-1 | Case control | 24 | 16-47 | unknown | 20 | SLEDAI  43 ± 7 | No |  | Serum | ELISA |
| **Ho et al. 2003**  ***USA*** | VCAM-1 | Case control | 73 | 39 (20-67) | 12.4 / 9.0 years (RSLE/SLE) | 28 | SLEDAI  RSLE 7.9±5.9 (range 0–20)  SLE 2.8±5.6 (range 0–32) | No |  | Plasma | ELISA |
|  | Thrombomodulin |  |  |  |  |  |  |  |  |  |  |
| **Robak et al. 2003**  ***Poland*** | VEGF | Cross-sectional | 52 | 41 (17-76) | 62 months | 20 | SLAM  N.M. | No |  | Serum | ELISA |
| **Wais et al. 2003**  ***Switzerland*** | Neopterin | Case control | 57 | 45 (22-77) | 10.5 years | 17 | BILAG  39 pts inactive disease (total BILAG score ≤ 5), 18 pts active  SLE. No means/medians | No |  | Plasma | ELISA |
|  | VCAM-1 |  |  |  |  |  |  |  |  |  |  |
|  | E-Selectin |  |  |  |  |  |  |  |  |  |  |
|  | ICAM-1 |  |  |  |  |  |  |  |  |  |  |
| **Boehme et al. 2004**  ***Germany*** | Thrombomodulin | Case control | 28 | 35 (18-65) | unknown | 10 | SLAM | No |  | Serum | ELISA |
| **El-Gamal et al. 2004**  ***Egypt*** | Thrombomodulin | Case control | 50 | 14 (5-18) | unknown | 30 | SLEDAI  33.35 ± 19.94 | No |  | Serum | ELISA |
| **Yajima et al. 2005** | sFkn | Casecontrol | 53 | 36 (SD 1.8) | unknown | 28 | SLEDAI | Yes (14) | 2 | Serum | ELISA |
| **Asanuma et al. 2006**  ***USA*** | MCP-1 | Case control | 74 | 41 (SD 12.3) | 9.6 years (SD 8.2) | 85 | SLEDAI  3.6 ± 3.5 | No |  | Plasma | ELISA |
| **Mak et al. 2006**  ***China*** | Adrenomedullin | Case control | 60 | 36 (26.8-46.8) | 8.7 years | 23 | SLEDAI  5.3±3.9 (nephritis)vs 2.9±2.9 (non nephritis) | No |  | Plasma | ELISA |
| **Sabry et al. 2006**  ***Egypt*** | ICAM-1 | Case control | 40 | 25 (17.9-33.46) | 25 months | 20 | SLEDAI  Active: 26.67 ± 5.02  Non-active: 9.75 ± 1.91 | No |  | Serum | ELISA |
| **Heshmat et al. 2007**  ***Egypt*** | VEGF | Case control | 25 | 14 (9-18) | unknown | 30 | SLEDAI  N.M. | No |  | Serum | ELISA |
| **Kuryliszyn-Moskal et al. 2007**  ***Poland*** | VEGF | Case control | 47 | 40 (20-71) | 8.1 years | 30 | SLEDAI  11.2±4.5 | No |  | Serum | ELISA |
| **Curiel et al. 2008**  ***USA*** | vWF | Prospective observational study | 40 | 37 (20-53) | 8,76 years | - | SLEDAI  Mean: 4.78 (0-16) Median: 2 | Yes (40) |  | Plasma | ELISA |
| **Ibrahim et al. 2008**  ***Egypt*** | VEGF | Case control | 30 | 25 (14-45) | unknown | 10 | SLEDAI  N.M. | No |  | Serum | ELISA |
| **Nienhuis et al. 2008**  ***The Netherlands*** | VCAM-1 | Case control | 10 | 31 (22-41) | unknown | 10 | SLEDAI  N.M. | Yes (10) | 2 | Serum | ELISA |
|  | sRAGE |  |  |  |  |  |  |  |  |  |  |
| **Akbarian et al. 2009**  ***Iran*** | Thrombomodulin | Case control | 64 | 32 (19-56) | 71.33 months | 27 active LN, 21 active SLE without renal involv. | SLEDAI  N.M. | No |  | Serum | ELISA |
| **Batuca et al. 2009**  ***UK*** | VCAM-1 | Case control | 77 | 40 | unknown | 50 | BIlAG  4.5 ± 3.8 | No |  | Serum | ELISA |
|  | ICAM-1 |  |  |  |  |  |  |  |  |  |  |
| **Colombo et al. 2009**  ***Italy*** | VEGF | Case control | 80 | 43 (18-65) | 14.8 years | 80 | SLEDAI  68.8% had a SLEDAI  score 2 and 31.3% a SLEDAI score 4 | No |  | Serum & Plasma | ELISA |
| **Elhelaly et al. 2009**  ***Egypt*** | VEGF | Case control | 23 | 8-18 | unknown | 25 | SLAM  N.M. | No |  | Serum | ELISA |
| **Hrycek et al. 2009**  ***Poland*** | VEGF | Cross sectional | 40 | 47 (SD 14) | Months-years | 24 | SLEDAI  N.M. | No |  | Serum | ELISA |
|  | HGF |  |  |  |  |  |  |  |  |  |  |
|  | bFGF |  |  |  |  |  |  |  |  |  |  |
| **Kümpers et al. 2009**  ***Germany*** | Angiopoietin-1 | Case control | 43 | 45 (21-67) | unknown | 30 | SLEDAI  2 (1-9) | No |  | Serum | ELISA |
|  | Angiopoietin-2 |  |  |  |  |  |  |  |  |  |  |
|  | VCAM-1 |  |  |  |  |  |  |  |  |  |  |
| **Kuryliszyn-Moskal et al. 2009**  ***Poland*** | VEGF | Case control | 76 | 40 (19-72) | 8.2 years | 34 | SLEDAI  9.3 ± 6.2 | No |  | Serum | ELISA |
|  | Endothelin-1 |  |  |  |  |  |  |  |  |  |  |
|  | E-Selectin |  |  |  |  |  |  |  |  |  |  |
|  | Thrombomodulin |  |  |  |  |  |  |  |  |  |  |
| **De Leeuw et al. 2009**  ***The Netherlands*** | VCAM-1 | Prospective observational study | 74 | 37 ( ± 14) | 5-10 years | 74 | SLEDAI  2 (0–2) | Yes (52) |  | Serum | ELISA |
|  | Thrombomodulin |  |  |  |  |  |  |  |  |  |  |
|  | vWF |  |  |  |  |  |  |  |  |  |  |
| **Alzawawy et al. 2009**  ***Egypt*** | MCP-1 | Case control | 30 | 25 (15-42) | 52.4 (5-120) months | 10 | SLEDAI  N.M. (only percentages) | No |  | Serum & urine | ELISA |
| **Kong et al. 2009**  ***Singapore*** | IP-10 | Case control | 464 | 40 (SD 14.7) | 113 months (SD 93. | 50 | SLAM  N.M. Only percentages | Yes (44) |  | Serum & PBMC | ELISA |
| **Reynolds et al. 2010**  ***USA*** | E-Selectin | Case cotrol | 119 | 43 (SD 12.4) | unknown | 71 | SLEDAI  N.M. Only percentages | No |  | unknown | ELISA |
|  | Adiponectin |  |  |  |  |  |  |  |  |  |  |
| **Ciolkiewicz et al. 2010**  ***Poland*** | VEGF | Cross-sectional | 80 | 40 (± 13.3) | Mean 7.9 (SD 7.2) years | - | SLEDAI  9.3 (2 to 26) | No |  | Serum | ELISA |
|  | E-Selectin |  |  |  |  |  |  |  |  |  |  |
|  | TM |  |  |  |  |  |  |  |  |  |  |
|  | Endothelin-1 |  |  |  |  |  |  |  |  |  |  |
| **Elwy et al. 2010**  ***Egypt*** | VCAM-1 | Cross-sectional | 43 | 41 (SD 13.2) | Mean 10.7 (SD 9.4) years | 20 | BILAG  N.M. Only percentages | Yes (22) |  | Serum | ELISA |
|  | ICAM-1 |  |  |  |  |  |  |  |  |  |  |
|  | Neopterin |  |  |  |  |  |  |  |  |  |  |
| **Shah et al. 2011**  ***India*** | MCP | Case control | 30 | 27 ( ± 7.48) | 5.1 (SD2.2) years | 30 | SLEDAI  35.56 ± 16.31 | No |  | Plasma & serum | ELISA |
|  | IP-10 |  |  |  |  |  |  |  |  |  |  |
| **Bassyouni et al. 2012**  ***Egypt*** | Endoglin | Case control | 86 | 30 (± 9.11) | 6.19 (SD 4.54) years | 36 | SLEDAI  7.99 ± 7.94 | No |  | Serum | ELISA |
| **Gu et al. 2012**  ***China*** | s-GITRL | Case control | 58 | 31 (11.5) | 24.9 (SD 40.9) years | 30 | SLEDAI  (22.5 ± 12.02) | No |  | Serum | ELISA |
| **Moneib et al. 2012**  ***Egypt*** | VEGF | Case control | 30 | 29 (± 10.2) | 2-8 years | 15 | No correlation study perfomed | No |  | Serum | ELISA |
| **Salama et al. 2012**  ***Egypt*** | Angiopoietin-2 | Case control | 50 | 29 ( ± 8) | 5.5 (SD3.59) years | 30 | SLEDAI  N.M. only percentages | No |  | Plasma | ELISA |
|  | P-Selectin |  |  |  |  |  |  |  |  |  |  |
| **Santos et al. 2012**  ***Portugal*** | VCAM-1 | Case control | 127 | 44 (SD13.9) | 8.4 (SD6.5) years | 124 | SLEDAI  3.46 ± 4.5 | No |  | Serum | ELISA |
|  | ICAM-1 |  |  |  |  |  |  |  |  |  |  |
|  | Thrombomodulin |  |  |  |  |  |  |  |  |  |  |
| **Robak et al. 2013**  ***Poland*** | VEGF, | Case control | 60 | 39 (21-66) | 66 months (mean) | 20 | SLEDAI  N.M> | No |  | Serum | ELISA |
|  | PlGF |  |  |  |  |  |  |  |  |  |  |
| **Zhou et al. 2014**  ***China*** | VEGF | Case control | 54 | 37 (± 12.52) | unknown | 28 | SLEDAI  N.M., only percentages | No |  | Serum | ELISA |
|  | bFGF |  |  |  |  |  |  |  |  |  |  |
|  | PlGF |  |  |  |  |  |  |  |  |  |  |
| **Skeoch et al. 2014**  ***UK*** | E-Selectin | Case control | 178 | 53 (46-61) | 13 (7-23) | 69 | SLEDAI  2 (0, 4) | No |  | Plasma | ELISA |
|  | VCAM-1 |  |  |  |  |  |  |  |  |  |  |
| **Bărbulescu et al. 2015**  ***Romania*** | VEGF | Cross sectional | 18 | 45 (20-66) | 8.00 ± 5.54 years | 17 | SLEDAI  7.55 ± 4.06 | No |  | Serum | Human VEGF Assay kit-IBL |
| **Assandri et al. 2015**  ***Italy*** | Pentraxin-3 | Case control | 64 | 46 (SD 15.1) | unknown | 60 | SLEDAI  8 ± 3.5 (0-16) | Yes (2) | 2 | Plasma | ELISA |
| **Cieslik et al. 2015**  ***Poland*** | Pentraxin-3 | Case Control | 56 | 41 (± 14.3) | 5.89 ± 5.63 years | 28 | SLEDAI  6.77 ± 1.44 | No |  | Serum | ELISA |
|  | VCAM-1 |  |  |  |  |  |  |  |  |  |  |
|  | vWF |  |  |  |  |  |  |  |  |  |  |
|  | Thrombomodulin |  |  |  |  |  |  |  |  |  |  |
|  | MCP-1 |  |  |  |  |  |  |  |  |  |  |
|  | E-Selectin |  |  |  |  |  |  |  |  |  |  |
|  | P-Selectin |  |  |  |  |  |  |  |  |  |  |
|  | ICAM-1 |  |  |  |  |  |  |  |  |  |  |
|  | PECAM-1 |  |  |  |  |  |  |  |  |  |  |
| **Martin-Rodriguez et al. 2015**  ***Spain*** | ADAMTS13 | Cross sectional | 50 | 16-58 | unknown | 50 | SLEDAI  6 .64 ± 4 (2–21) | No |  | Plasma | ELISA |
|  | vWF |  |  |  |  |  |  |  |  |  |  |
|  | VCAM-1 |  |  |  |  |  |  |  |  |  |  |
| **El-Serougy et al. 2015**  ***Egypt*** | Adrenomedullin | Cross sectional | 60 | 28 (19-36) | 5.5 +/- 4.5  years | 20 | SLEDAI  Nephritis: 13.8 ± 7.1 (2–30)  No nephritis: 7.4 ± 6.7 (2–27) | No |  | Serum | ELISA |
| **Skare et al. 2015**  ***Brazil*** | Pentraxin-3 | Case control | 92 | 41 (29-47.5) | 101.1 (SD 60.8) months | 94 | SLEDAI  N.M. | No |  | Plasma | ELISA |
| **Robak et al. 2009**  ***Poland*** | VEGF | Case control | 61 | 39 (21-66) | 8m-20 years (mean 64 m) | 20 | SLAM  N.M. | No |  | Serum | ELISA |
|  | PlGF |  |  |  |  |  |  |  |  |  |  |
|  | VCAM-1 |  |  |  |  |  |  |  |  |  |  |
|  | Endoglin |  |  |  |  |  |  |  |  |  |  |
| **Kim et al. 2013**  ***South-Korea*** | GAS6 | Cross sectional | 150 | 36 (24-49) | 1.5+/-2.2 years | 50 | SLEDAI  7.8 ± 4.9 | Yes (50) | 2 | Serum | ELISA |
| **Liu et al.**  **2015**  ***China*** | VEGF | Case control | 75 | 35 ( ± 11.79) | unknown | 40 | SLEDAI  N.M.? | No |  | Serum | ELISA |
|  | bFGF |  |  |  |  |  |  |  |  |  |  |
|  | HGF |  |  |  |  |  |  |  |  |  |  |
|  | PlGF |  |  |  |  |  |  |  |  |  |  |
| **Abou-Raya et al. 2016**  ***Egypt*** | sRAGE | Case control | 35 | 34 (+/-11.53) | 6.43 +/- 2.08 years | 20 | SLEDAI  Only percentages | No |  | Serum | ELISA |
| **Al-Yasaky et al. 2005** | Thrombomodulin | Case control | 60 | 24 (20-39) | 6 months - 19 years | 30 | SLEDAI | No |  | Serum | ELISA |
|  | Adrenomedullin |  |  |  |  |  |  |  |  |  |  |
| **Suh et al. 2010**  ***USA/Korea*** | Gas6 | Case control | 107 | 40 ( ± 15.5) | unknown | 45 | SLEDAI & BILAG  SLEDAI: 6.02 ± 4.3  BILAG: 6.93 ± 5.34 | No |  | Plasma | ELISA |
| **Ekman et al. 2011**  ***Sweden*** | Gas6 | Case control | 96 | 45 (14-85) | 10 years | - | SLEDAI  8.7 (range 0–32) | No |  | Plasma | ELISA |
| **Shimada et al. 2014**  ***Japan*** | Pentraxin-3 | Case control | 65 | ? | 6 years | 53 | SLEDAI & BILAG  N.M. | No |  | Serum & plasma | ELISA |
| **Gupta et al. 2016**  ***India*** | MCP-1 | Case control | 121 | 27 | unknown | 58 | SLEDAI  N.A. | Yes (121) |  | Serum & Urine | ELISA |
| **Chung et al. 2016** | E-Selectin | Case control | 116 | 40 (SD 12) | unknown | 84 | No | No |  | unknown | ELISA |
|  | ICAM-1 |  |  |  |  |  |  |  |  |  |  |
|  | VCAM-1 |  |  |  |  |  |  |  |  |  |  |
| **Lewis et al. 2016**  ***UK*** | E-Selectin | Prospective observational study | 21 | 41 (IQR 34.3-51.3) | Follow up 16.5 months | - | ECLAM  N.M. | Yes (21) | 3,7 (0-4) | Serum | ELISA |
|  | P-Selectin |  |  |  |  |  |  |  |  |  |  |
|  | ICAM-1 |  |  |  |  |  |  |  |  |  |  |
|  | VCAM-1 |  |  |  |  |  |  |  |  |  |  |
| **Mahayidin et al. 2016**  ***Malaysia*** | VEGF | Cross sectional | 96 | 31 (± 8.86) | active 1 (2.75) vs non-active 6.5 (7.5) | 50 | SLEDAI  Only percentages | No |  | Serum | ELISA |
|  | ICAM-1 |  |  |  |  |  |  |  |  |  |  |
|  | VCAM-1 |  |  |  |  |  |  |  |  |  |  |
| **Sahin et al. 2017**  ***Turkey*** | Pentraxin-3 | Case control | 76 | 16 (SD 3.3) | 4.4 years | 41 | SLEDAI  10.3 ± 4.8 (at disease onset) to 5.2 ± 5.3 (at last examination). | No |  | Serum | ELISA |
| **Tydén et al. 2017**  ***Sweden*** | VCAM-1 | Case control | 148 | 48 (20-82) | unknown | 79 | No correlation measured | No |  | Plasma | ELISA |
| **Da Rosa Franchi Santos et al. 2018**  ***Brazil*** | ICAM-1 | Case control | 126 | 40 (SD 13.5) | unknown | 48 | SLEDAI | No |  | unknown | Luminex |
|  | VCAM-1 |  |  |  |  |  |  |  |  |  |  |
|  | E-Selectin |  |  |  |  |  |  |  |  |  |  |
|  | P-Slectin |  |  |  |  |  |  |  |  |  |  |
|  | PECAM-1 |  |  |  |  |  |  |  |  |  |  |
|  | PAI-1 |  |  |  |  |  |  |  |  |  |  |
| **Demir et al. 2018**  ***Turkey*** | ICAM-1 | Case control | 66 | 34 (SD 8.9) | 74 months | 28 | SLEDAI 1.15 ± 1.53 | No |  | Serum | ELISA |
|  | E-Selectin |  |  |  |  |  |  |  |  |  |  |
| **Hajialilo et al. 2018**  ***Iran*** | Endothelin-1 | Case control | 60 | 32 (SD 7.7) | 4.5 years | 40 | SLEDAI  8.75 ± 4.50 (range 2–22 | No |  | Serum | ELISA |
|  | VCAM-1 |  |  |  |  |  |  |  |  |  |  |
| **Zhang et al. 2018**  ***China*** | IP-10 | Case control | 46 | 13 (SD 2.6) | 32.4 months | 31 | SLEDAI  4.35 ± 3.35 | No |  | Serum | Luminex |
|  | Fas |  |  |  |  |  |  |  |  |  |  |
|  | FasL |  |  |  |  |  |  |  |  |  |  |
| **Živković et al. 2018**  ***Serbia*** | MCP-1 | Case control | 72 | 46 (SD 9.3) | 11.24 years | 30 | SLEDAI  10.9 ± 6.98 | No |  | Serum | ELISA |
| **Hardt et al. 2018**  ***Sweden*** | VCAM-1 | Case control | 398 / 225 USA cohort | 47 (SD 15) / unknown | unknown | 322 | No | Yes (19) |  | Serum | ELISA |
| **Adhya et al. 2019**  ***Qatar*** | VEGF | Cross sectional | 87 | 39 | unknown | - | SLEDAI  6.7 | No |  | Serum | Luminex |
|  | MCP-1 |  |  |  |  |  |  |  |  |  |  |
|  | IP-10 |  |  |  |  |  |  |  |  |  |  |
| **Alves et al. 2019**  ***Brazil*** | IP-10 | Case control | 46 | 40 (SD 9.6) | 10.8 years | other AIZ 44 / HC 8 | SLEDAI  5.02 (4.63) | No |  | Serum | Flowcytometry |
| **El-Gazzar et al. 2019**  ***Egypt*** | VEGF | Case control | 84 | 5 | unknown | 33 | SLEDAI  With APS: 6.8 ± 5.8  Without APS:  5.4 ± 4.4 | No |  | Serum | ELISA |
| **Hegazy et al. 2019** | ICAM-1 | Case control | 50 | 44 (SD 10.5) | 7.97 years | SSc 30 / HC 60 | Unknown | No |  | Serum | ELISA |
| **Lee et al. 2019**  ***Taiwan*** | Angiopoietin-1 | Case control | 118 | 20 (SD 6.3) | 7 years | 40 | SLEDAI  Only percentages | No |  | Serum | ELISA |
|  | Angiopoietin-2 |  |  |  |  |  |  |  |  |  |  |
|  | Tie2 |  |  |  |  |  |  |  |  |  |  |
|  | Thrombomodulin |  |  |  |  |  |  |  |  |  |  |
|  | VEGF |  |  |  |  |  |  |  |  |  |  |
|  | ADAMTS13 |  |  |  |  |  |  |  |  |  |  |
| **Ramirez et al. 2019**  ***Italy*** | Pentraxin-3 | Case control | 55 | 12 | 12 years | HC 79/ GPA 38 / RA 21 | BILAG & SLEDAI.  N.M. | No |  | Serum | ELISA |
| **Wojdasiewicz et al. 2019** | VEGF | Case control | 28 | 34 (19-74) | 2 years | 42 / 35 / 60 | No | No |  | Serum | ELISA |
|  | BAFF |  |  |  |  |  |  |  |  |  |  |
| **Mirioglu et al. 2020**  ***Turkey*** | TWEAK | Case control | 61 | 35 (IQR 26.3-38) | 78.6 months | 20 | SLEDAI  Active: 11.4 (6.1)  Inactive:  0.7 (0.9) | No |  | Serum | ELISA |
|  | NGAL |  |  |  |  |  |  |  |  |  |  |
|  | MCP-1 |  |  |  |  |  |  |  |  |  |  |
| **Cicarini et al. 2020**  ***Brazil*** | Thrombomodulin | Case control | 60 | 40 (SD 13.7) | 8.5 years | 30 | SLEDAI  4.50 (0–18) | No |  | Plasma | ELISA |
|  |  |  |  |  |  |  |  |  |  |  |  |

*Age patients in years, median (range) or, when mentioned; (SD)

**Different disease activity measurements: BILAG (British Isles Lupus Assessment Group), SLEDAI (Systemic Lupus Erythematosus Disease Activity Index), ECLAM: European Consensus Lupus Activity Measurement, SLAM(-R): Systemic Lupus Activity Measure(-Revised)

**
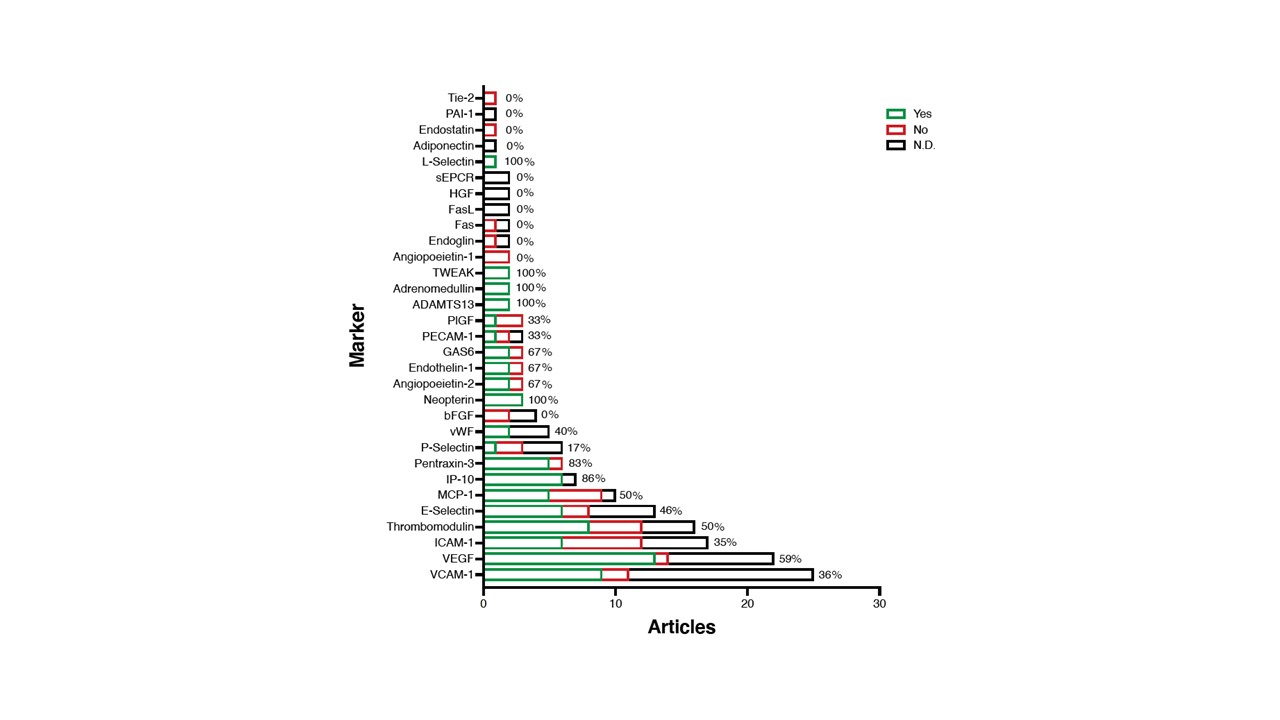
**

**Figure A**. **Overview of the number of articles per EC marker**

Overview of the number of articles per EC marker reporting whether or not a significant correlation (yes (green box), no (red box), not determined (N.D.) (black box)) between plasma/serum levels of each EC marker and validated SLE disease activity index (i.e. SLEDAI, BILAG, SLAM, ECLAM) was given. The percentage indicates the number of articles per EC marker in which a significant correlation with disease activity was reported (% ‘yes’ of total citations). Correlation was tested with spearman’s rank or Pearson’s correlation coefficients
