# Supplementary material for: Dysregulated endothelial cell markers in systemic lupus erythematosus: a systematic review and meta-analysis
Source: J Inflamm (Lond). 2023 May 16;20:18. doi: 10.1186/s12950-023-00342-1 (PMC10189957; doi:10.1186/s12950-023-00342-1)
Supplement: Supplementary file 5 — Additional file 5. Meta-analyses per marker. Figures S5A-S5L. Spearman’s rho correlations. Figures S5M-S5P. Pearson’s r correlations. [file 12950_2023_342_MOESM5_ESM.docx]

**Supplementary File 5**

*For calculations, the Meta-Essentials tool by Erasmus Research Institute and of Management (ERIM) was used. (1). Fixed effect models were used. Additionally, this method suggests the ‘weighted variance method’ for the calculation of a confidence interval (CI) for the overall correlation coefficient, based on a t(k) distribution (k = degrees of freedom, #studies -1) Calculated p-values are based on Fisher's Z Transformation*

Figures S5A-S5L: Spearman’s rho correlations

Figures S5M-S5P: Pearson’s r correlations

**Meta-analyses for Spearman’s rho correlations**

**Figure S5A**

VEGF: Meta-analysis of the correlation between VEGF and SLE disease activity

The correlation meta-analysis between VEGF levels and SLE disease activity was based on six studies (2-7), including a total of 390 patients (Fig. S5A). The overall correlation coefficient was significant (ρ = 0.30, 95%CI = [0.17, 0.41], p = 0.0020).


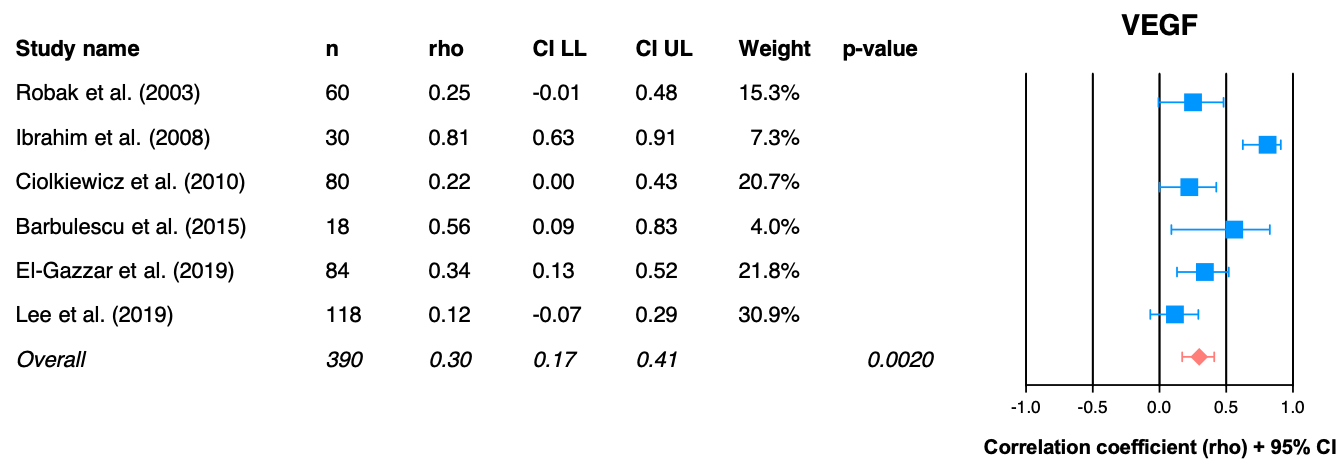


**Figure S5B**

Thrombomodulin: Meta-analysis of the correlation between Thrombomodulin and SLE disease activity

The correlation meta-analysis between Thrombomodulin levels and SLE disease activity was based on five studies (4, 7-10), including a total of 372 patients (Fig. S5B). The overall correlation coefficient was significant (ρ = 0.28, 95%CI = [0.14, 0.41], p = 0.0052).


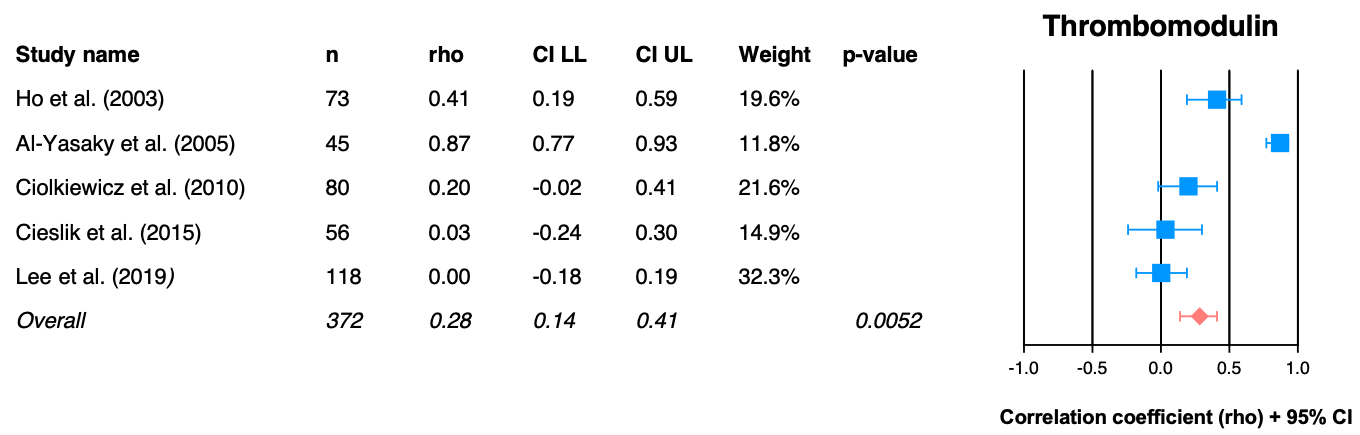


**Figure S5C**

ICAM-1: Meta-analysis of the correlation between ICAM-1 and SLE disease activity

The correlation meta-analysis between ICAM-1 levels and SLE disease activity was based on six studies (10-15), including a total of 386 patients (Fig. S5C). The overall correlation coefficient was significant (ρ = 0.27, 95%CI = [0.14, 0.38], p = 0.0035).


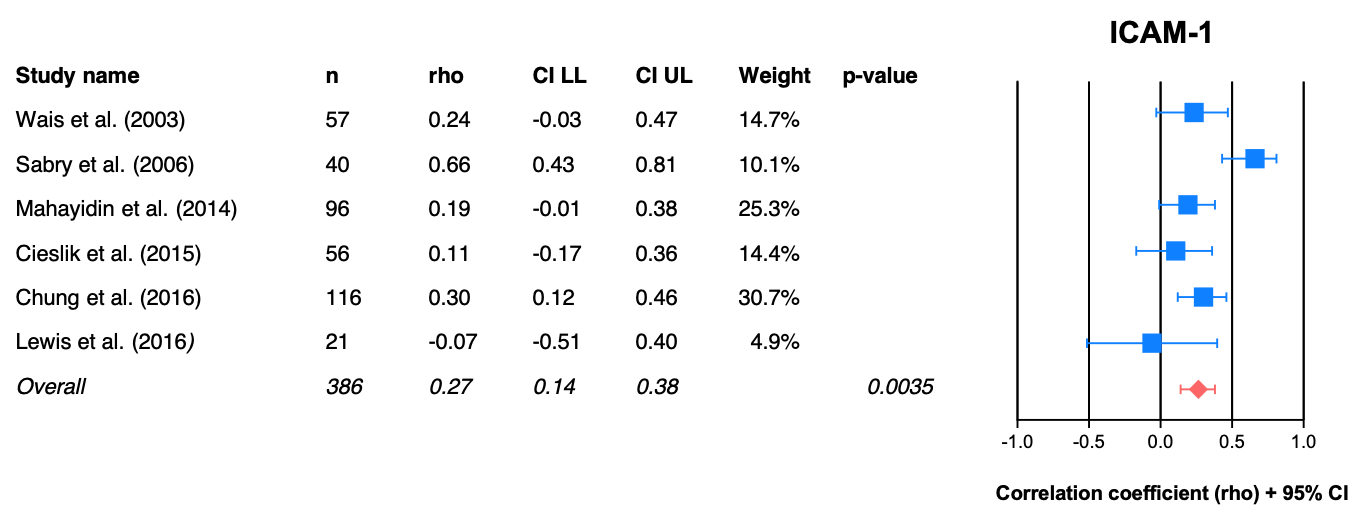


**Figure S5D**

VCAM-1: Meta-analysis of the correlation between VCAM-1 and SLE disease activity

The correlation meta-analysis between VCAM-1 levels and SLE disease activity was based on six studies (8, 10, 11, 13, 15, 16), including a total of 430 patients (Fig. S5D). The overall correlation coefficient was non-significant (ρ = 0.28, 95%CI = [0.16, 0.39], p = 0.0021).


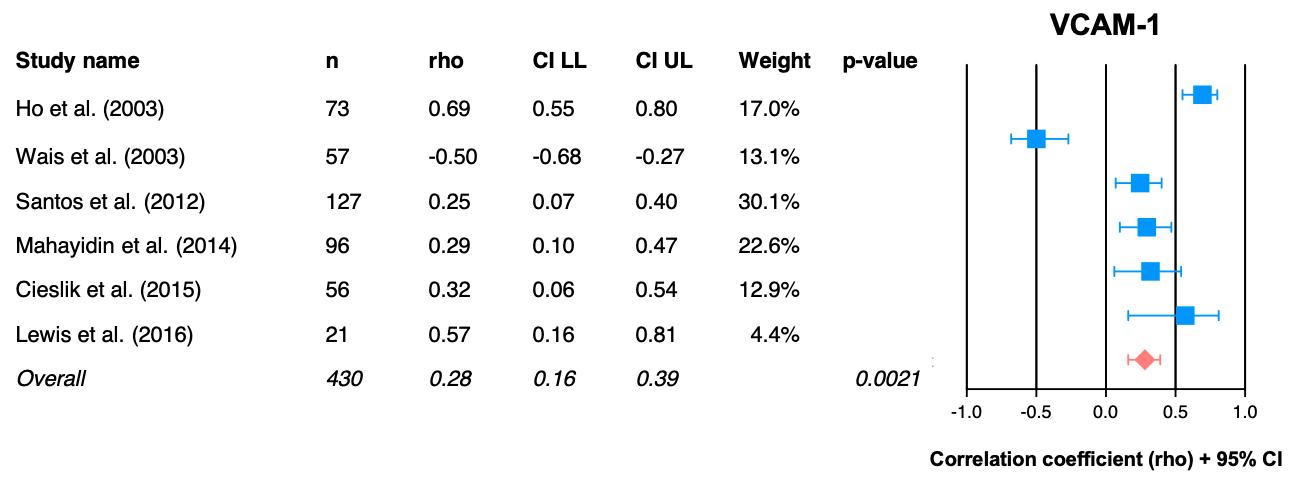


**Figure S5E**

MCP-1: Meta-analysis of the correlation between MCP1-1 and SLE disease activity

The correlation meta-analysis between MCP-1 levels and SLE disease activity was based on eight studies (10, 17-23), including a total of 514 patients (Fig. S5E). The overall correlation coefficient was significant (ρ = 0.24, 95%CI = [0.14, 0.34], p = 0.0010).


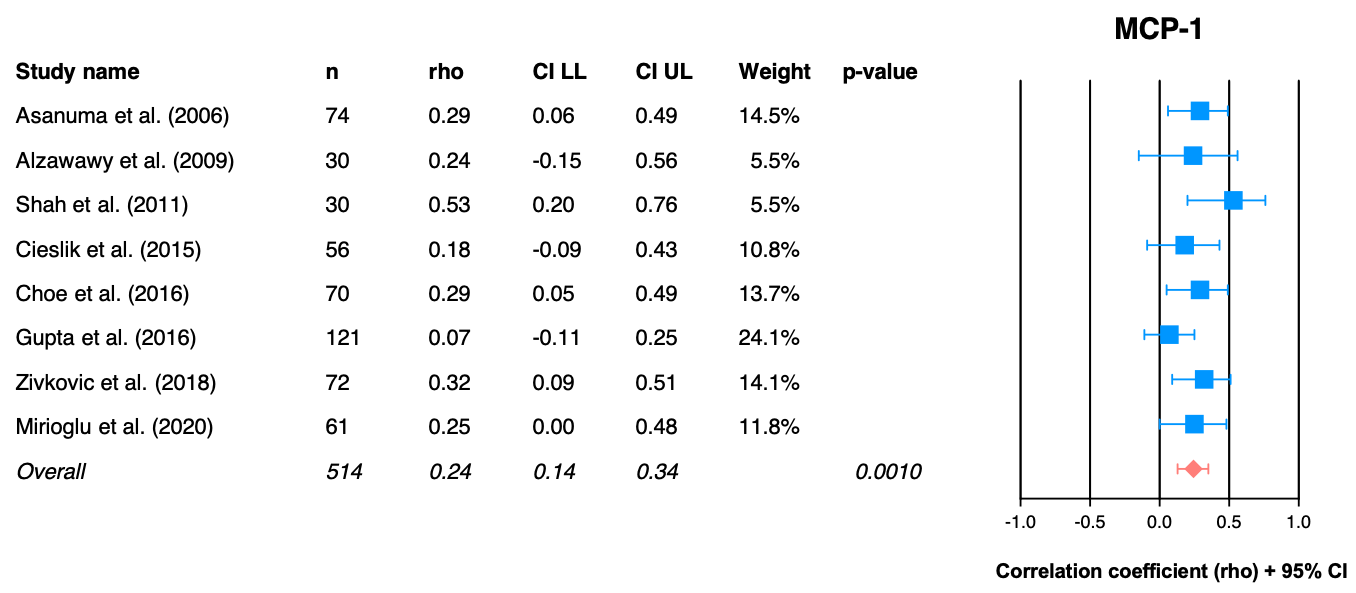


**Figure S5F**

E-Selectin: Meta-analysis of the correlation between E-Selectin and SLE disease activity

The correlation meta-analysis between E-Selectin levels and SLE disease activity was based on five studies (4, 10, 11, 14, 15), including a total of 330 patients (Fig. S5F). The overall correlation coefficient was non-significant (ρ = 0.12, 95%CI = [-0.03, 0.27], p = 0.097).


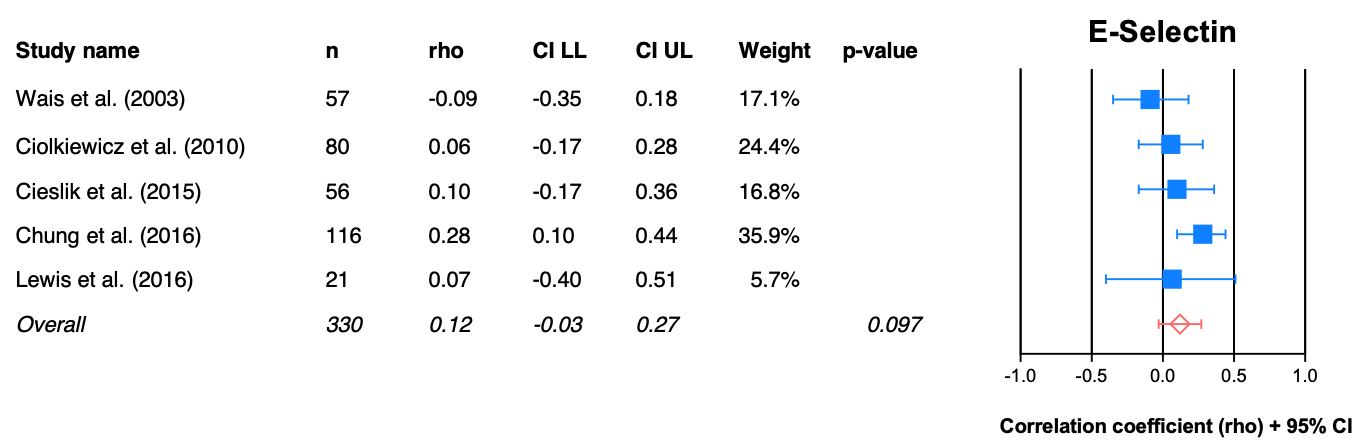


**Figure S5G**

Pentraxin-3: Meta-analysis of the correlation between Pentraxin-3 and SLE disease activity

The correlation meta-analysis between Pentraxin-3 levels and SLE disease activity was based on five studies (10, 24-27), including a total of 316 patients (Fig. S5G). The overall correlation coefficient was significant (ρ = 0.51, 95%CI = [0.38, 0.62], p = 0.0006).


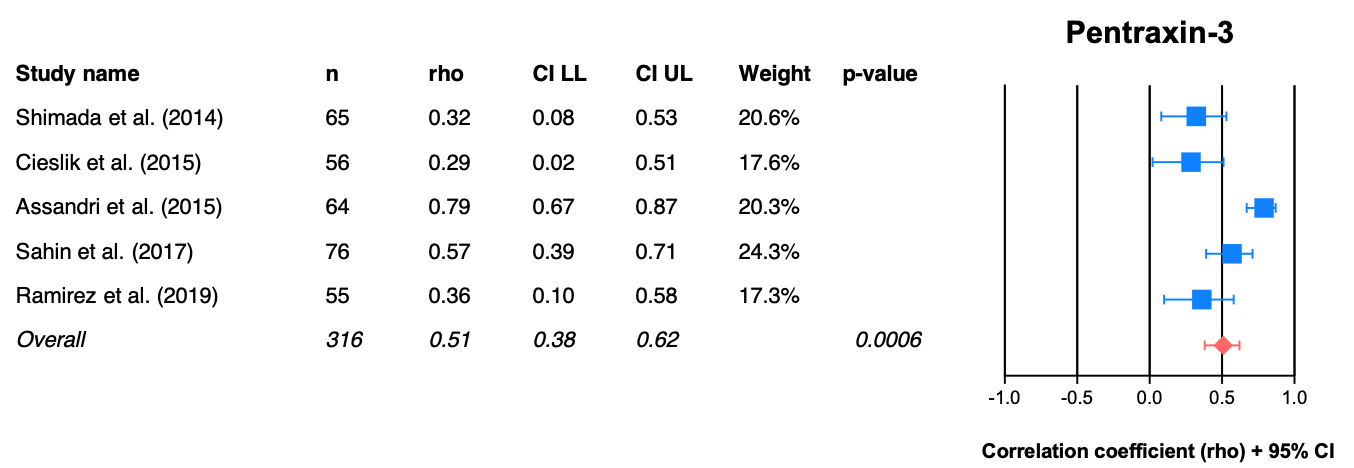


**Figure S5H**

IP-10: Meta-analysis of the correlation between IP-10 and SLE disease activity
The correlation meta-analysis between IP-10 levels and SLE disease activity was based on five studies (19, 20, 28-30), including a total of 656 patients (Fig. S5H). The overall correlation coefficient was non-significant (ρ = 0.20, 95%CI = [0.10, 0.31], p = 0.0064).


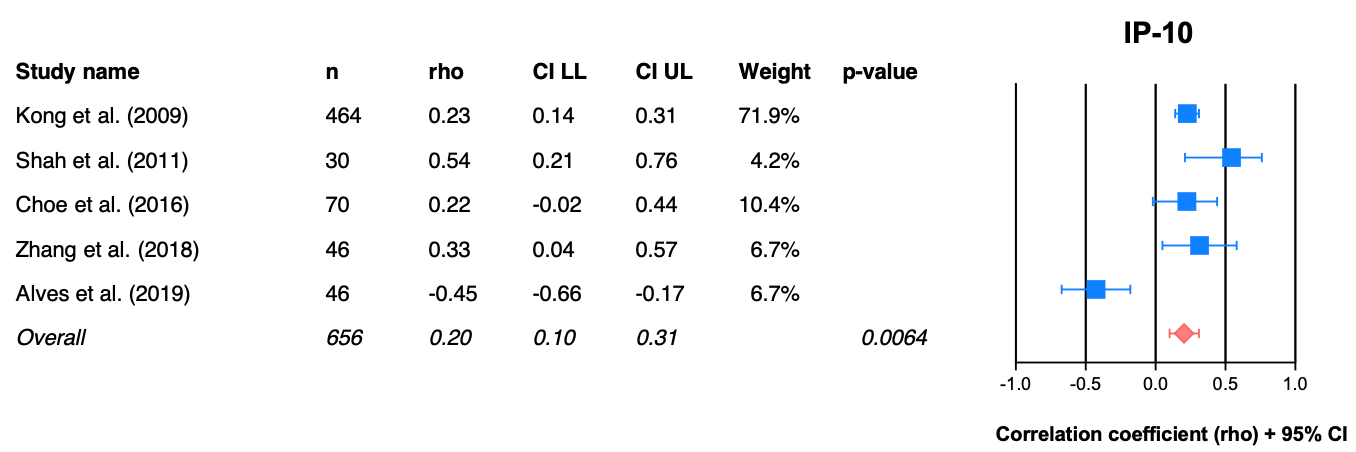


**Figure S5I**

Angiopoietin-2: Meta-analysis of the correlation between Angiopoietin-2 and SLE disease activity
The correlation meta-analysis between Angiopoietin-2 levels and SLE disease activity was based on two studies (7, 31), including a total of 161 patients (Fig. S5I). The overall correlation coefficient was non-significant (ρ = 0.28, 95%CI = [-0.63, 0.86], p = 0.17).


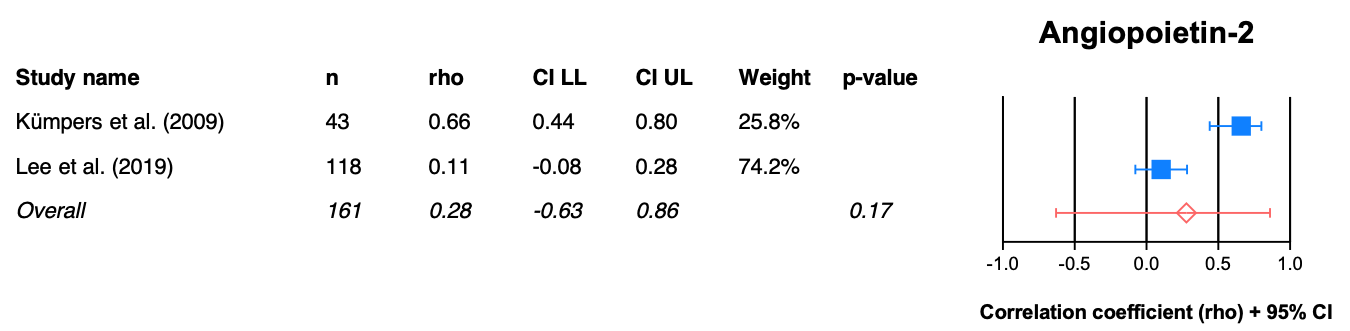


**Figure S5J**

vWF: Meta-analysis of the correlation between vWF and SLE disease activity
The correlation meta-analysis between vWF levels and SLE disease activity was based on two studies (10, 32), including a total of 96 patients (Fig. S5J). The overall correlation coefficient was non-significant (ρ = 0.45, 95%CI = [-0.69, 0.95], p = 0.14).


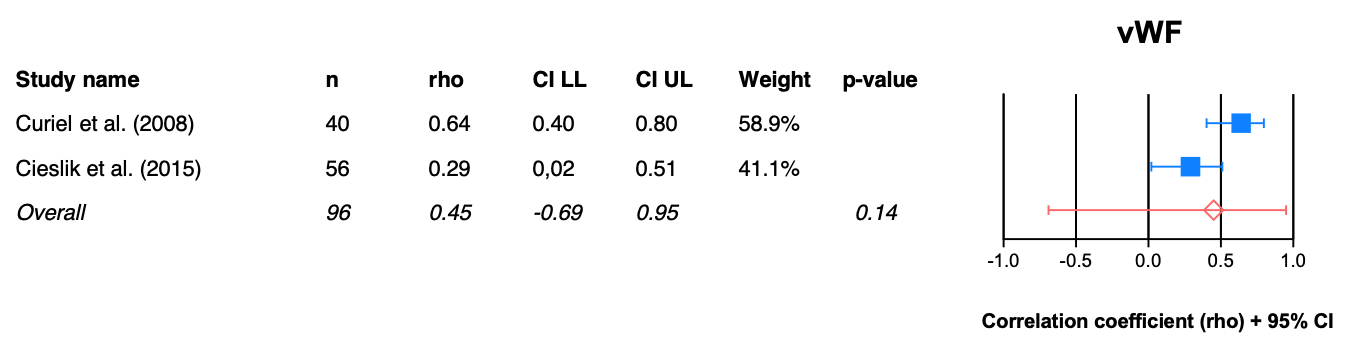


**Figure S5K**

P-Selectin: Meta-analysis of the correlation between P-Selectin and SLE disease activity

The correlation meta-analysis between P-Selectin levels and SLE disease activity was based on two studies (10, 15), including a total of 77 patients (Fig. S5K). The overall correlation coefficient was non-significant (ρ = 0.09, 95%CI = [-0.89, 0.92], p = 0.60).


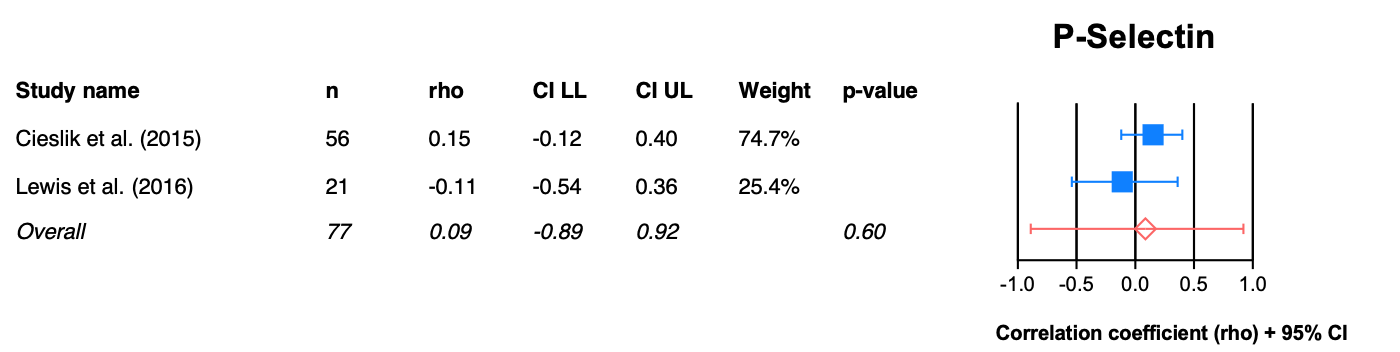


**Figure S5L**

TWEAK: Meta-analysis of the correlation between TWEAK and SLE disease activity
The correlation meta-analysis between TWEAK levels and SLE disease activity was based on two studies (20, 23), including a total of 131 patients (Fig. S5L). The overall correlation coefficient was non-significant (ρ = 0.31, [-0.67, 0.90], p = 0.17).


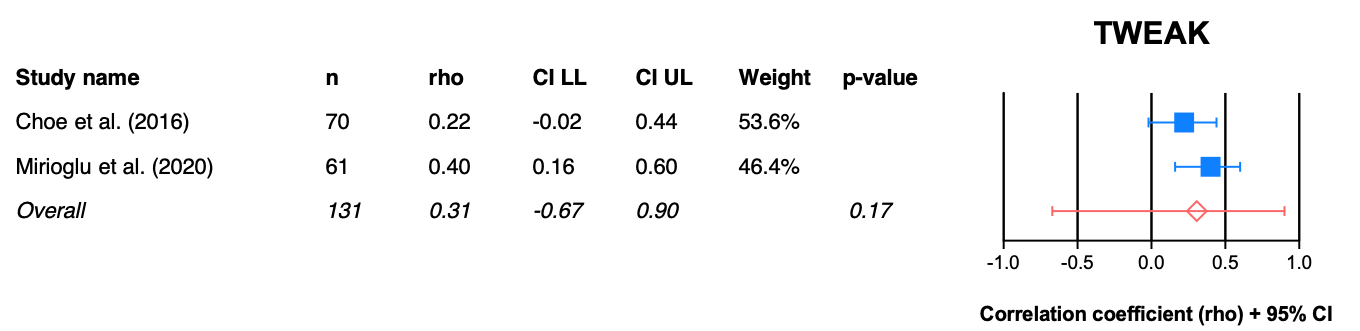


**Meta-analyses for Pearon’s r correlations**

**Figure S5M**

VEGF : Meta-analysis of the correlation between VEGF and SLE disease activity

The correlation meta-analysis between VEGF levels and SLE disease activity was based on two studies (33, 34), including a total of 79 patients (Fig. S5M). The overall correlation coefficient was non-significant (r = 0.59, 95%CI = [-0.67, 0.97], p = 0.11).


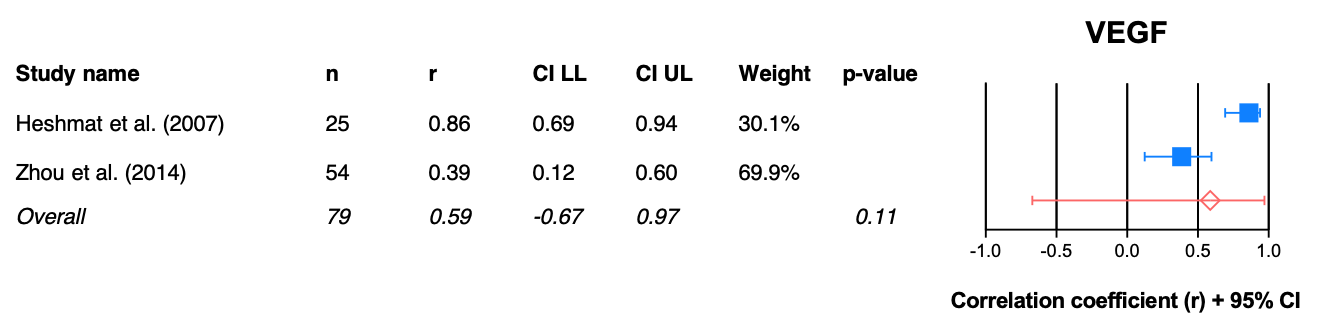


**Figure S5N**Thrombomodulin: Meta-analysis of the correlation between Thrombomodulin and SLE disease activity
The correlation meta-analysis between Thrombomodulin levels and SLE disease activity was based on three studies (35-37), including a total of 146 patients (Fig. S5N). The overall correlation coefficient was significant (r = 0.59, 95%CI = [0.31, 0.78], p = 0.015).


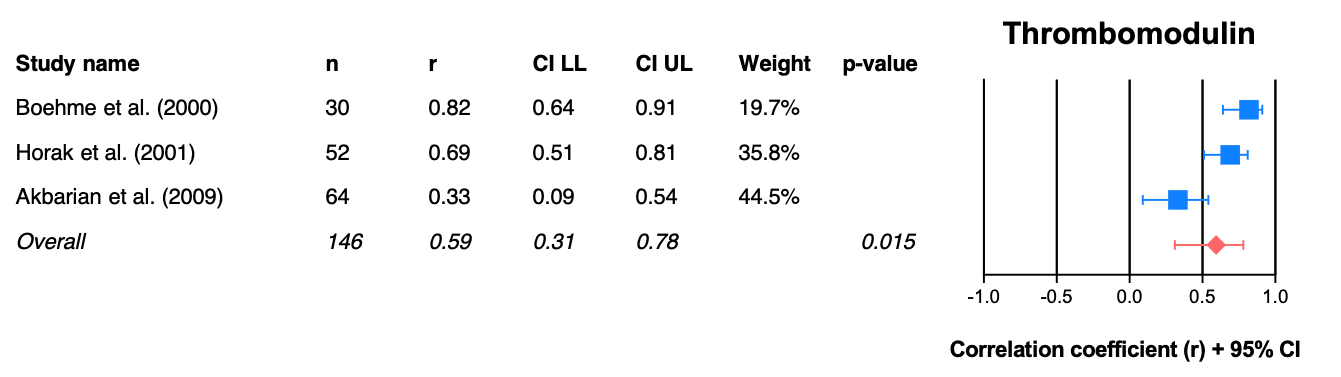


**Figure S5O**

ICAM-1: Meta-analysis of the correlation between ICAM-1 and SLE disease activity

The correlation meta-analysis between ICAM-1 levels and SLE disease activity was based on three studies(35, 36, 38), including a total of 106 patients (Fig. S5O). The overall correlation coefficient was significant (r = 0.48, 95%CI = [0.08, 0.74] p = 0.036).


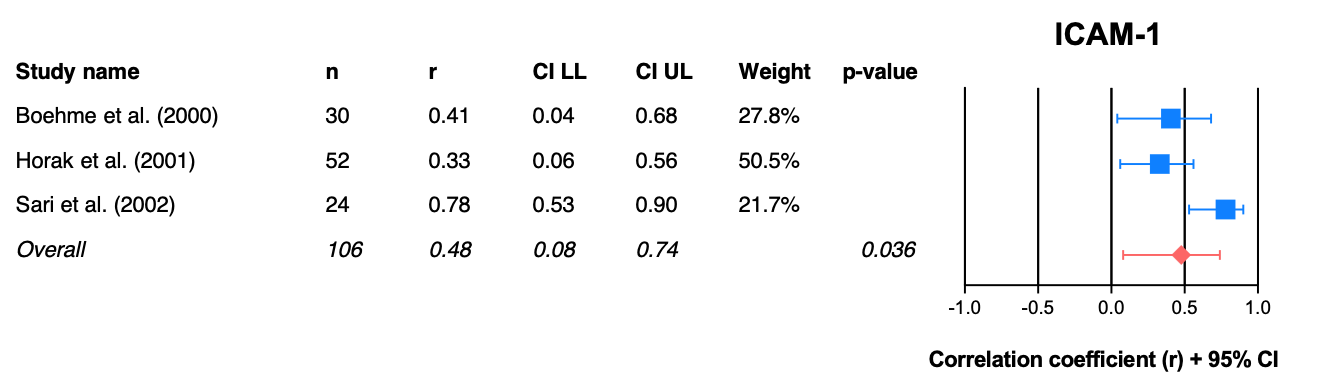


**Figure S5P**

VCAM-1: Meta-analysis of the correlation between VCAM-1 and SLE disease activity

The correlation meta-analysis between VCAM-1 levels and SLE disease activity was based on two studies (35, 36), including a total of 82 patients (Fig. S5P). The overall correlation coefficient was non-significant (r = 0.46, 95%CI = [-0.75, 0.96], p = 0.15).


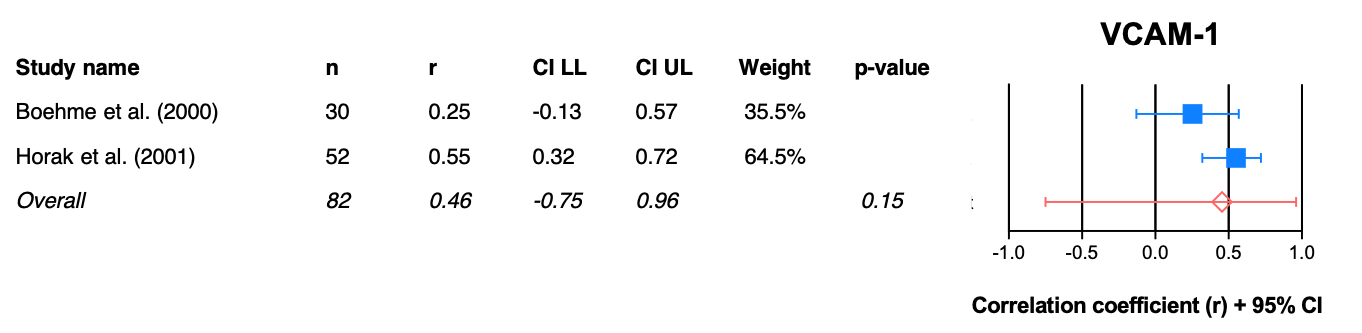


**References S5**

1. Suurmond R, van Rhee H, Hak T. Introduction, comparison, and validation of Meta-Essentials: A free and simple tool for meta-analysis. Res Synth Methods. 2017;8(4):537-53.

2. Robak E, Sysa-Jedrzejewska A, Robak T. Vascular endothelial growth factor and its soluble receptors VEGFR-1 and VEGFR-2 in the serum of patients with systemic lupus erythematosus. Mediators Inflamm. 2003;12(5):293-8.

3. Fakhry F. Ibrahim HMDaHHAS. Serum Levels of Vascular Endothelial Growth Factor and Hemoglobin Dielectric Properties in Patients with Systemic Lupus Erythematosus. Journal of Medical Sciences2008.

4. Ciolkiewicz M, Kuryliszyn-Moskal A, Klimiuk PA. Analysis of correlations between selected endothelial cell activation markers, disease activity, and nailfold capillaroscopy microvascular changes in systemic lupus erythematosus patients. Clin Rheumatol. 2010;29(2):175-80.

5. Bărbulescu AL, Vreju AF, Bugă AM, Sandu RE, Criveanu C, Tudoraşcu DR, et al. Vascular endothelial growth factor in systemic lupus erythematosus - correlations with disease activity and nailfold capillaroscopy changes. Rom J Morphol Embryol. 2015;56(3):1011-6.

6. El-Gazzar II, Ibrahim SE, El-Sawy WS, Fathi HM, Eissa AH. Assessment of vascular endothelial growth factor in systemic lupus erythematosus patients with anti-phospholipid syndrome. The Egyptian Rheumatologist. 2019;41(1):41-5.

7. Lee WF, Wu CY, Yang HY, Lee WI, Chen LC, Ou LS, et al. Biomarkers associating endothelial Dysregulation in pediatric-onset systemic lupus erythematous. Pediatr Rheumatol Online J. 2019;17(1):69.

8. Ho CY, Wong CK, Li EK, Tam LS, Lam CW. Elevated plasma concentrations of nitric oxide, soluble thrombomodulin and soluble vascular cell adhesion molecule-1 in patients with systemic lupus erythematosus. Rheumatology (Oxford). 2003;42(1):117-22.

9. Al-Yasaky AZ. Soluble thrombomoduline (STM) and human adrenomedullin (AM) in systemic lupus erythematosus and their relation to disese activity and renal affection In: Hala Mahfouz* MMaNZ, editor. Egypt Rheumatol Rehab2005.

10. Cieślik P, Hrycek A. Pentraxin 3 as a biomarker of local inflammatory response to vascular injury in systemic lupus erythematosus. Autoimmunity. 2015;48(4):242-50.

11. Wais T, Fierz W, Stoll T, Villiger PM. Subclinical disease activity in systemic lupus erythematosus: immunoinflammatory markers do not normalize in clinical remission. J Rheumatol. 2003;30(10):2133-9.

12. Sabry AA, Elbasyouni SR, Kalil AM, Abdel-Rahim M, Mohsen T, Sleem A. Markers of inflammation and atherosclerosis in Egyptian patients with systemic lupus erythematosus. Nephrology (Carlton). 2006;11(4):329-35.

13. Mahayidin H, Yahya NK, Wan Ghazali WS, Mohd Ismail A, Wan Ab Hamid WZ. The Usefulness of Endothelial Cell Adhesion Molecules and Anti-C1q Antibody in Monitoring Systemic Lupus Erythematosus Disease Activity. Int Sch Res Notices. 2014;2014:275194.

14. Chung CP, Ormseth MJ, Connelly MA, Oeser A, Solus JF, Otvos JD, et al. GlycA, a novel marker of inflammation, is elevated in systemic lupus erythematosus. Lupus. 2016;25(3):296-300.

15. Lewis MJ, Vyse S, Shields AM, Zou L, Khamashta M, Gordon PA, et al. Improved monitoring of clinical response in Systemic Lupus Erythematosus by longitudinal trend in soluble vascular cell adhesion molecule-1. Arthritis Res Ther. 2016;18:5.

16. Santos MJ, Carmona-Fernandes D, Canhão H, Canas da Silva J, Fonseca JE, Gil V. Early vascular alterations in SLE and RA patients--a step towards understanding the associated cardiovascular risk. PLoS One. 2012;7(9):e44668.

17. Asanuma Y, Chung CP, Oeser A, Shintani A, Stanley E, Raggi P, et al. Increased concentration of proatherogenic inflammatory cytokines in systemic lupus erythematosus: relationship to cardiovascular risk factors. J Rheumatol. 2006;33(3):539-45.

18. Alzawawy A, Zohary M, Ablordiny M, Eldalie M. Estimation of monocyte-chemoattractantprotein-1 (Mcp-1) level in patients with lupus nephritis. Int J Rheum Dis. 2009;12(4):311-8.

19. Shah D, Wanchu A, Bhatnagar A. Interaction between oxidative stress and chemokines: possible pathogenic role in systemic lupus erythematosus and rheumatoid arthritis. Immunobiology. 2011;216(9):1010-7.

20. Choe J-Y, Kim S-K. Serum TWEAK as a biomarker for disease activity of systemic lupus erythematosus. Inflammation Research. 2016;65(6):479-88.

21. Gupta R, Yadav A, Aggarwal A. Longitudinal assessment of monocyte chemoattractant protein-1 in lupus nephritis as a biomarker of disease activity. Clin Rheumatol. 2016;35(11):2707-14.

22. Živković V, Cvetković T, Mitić B, Stamenković B, Stojanović S, Radovanović-Dinić B, et al. Monocyte chemoattractant protein-1 as a marker of systemic lupus erythematosus: an observational study. Rheumatol Int. 2018;38(6):1003-8.

23. Mirioglu S, Cinar S, Yazici H, Ozluk Y, Kilicaslan I, Gul A, et al. Serum and urine TNF-like weak inducer of apoptosis, monocyte chemoattractant protein-1 and neutrophil gelatinase-associated lipocalin as biomarkers of disease activity in patients with systemic lupus erythematosus. Lupus. 2020;29(4):379-88.

24. Shimada Y, Asanuma YF, Yokota K, Yoshida Y, Kajiyama H, Sato K, et al. Pentraxin 3 is associated with disease activity but not atherosclerosis in patients with systemic lupus erythematosus. Mod Rheumatol. 2014;24(1):78-85.

25. Assandri R, Monari M, Colombo A, Dossi A, Montanelli A. Pentraxin 3 Plasma Levels and Disease Activity in Systemic Lupus Erythematosus. Autoimmune Dis. 2015;2015:354014.

26. Sahin S, Adrovic A, Barut K, Durmus S, Gelisgen R, Uzun H, et al. Pentraxin-3 levels are associated with vasculitis and disease activity in childhood-onset systemic lupus erythematosus. Lupus. 2017;26(10):1089-94.

27. Ramirez GA, Rovere-Querini P, Blasi M, Sartorelli S, Di Chio MC, Baldini M, et al. PTX3 Intercepts Vascular Inflammation in Systemic Immune-Mediated Diseases. Front Immunol. 2019;10:1135.

28. Kong KO, Tan AW, Thong BY, Lian TY, Cheng YK, Teh CL, et al. Enhanced expression of interferon-inducible protein-10 correlates with disease activity and clinical manifestations in systemic lupus erythematosus. Clin Exp Immunol. 2009;156(1):134-40.

29. Zhang CX, Cai L, Shao K, Wu J, Zhou W, Cao LF, et al. Serum IP-10 is useful for identifying renal and overall disease activity in pediatric systemic lupus erythematosus. Pediatr Nephrol. 2018;33(5):837-45.

30. Alves LCV, Carvalho MG, Nunes FFC, Reis EA, Ferreira GA, Calderaro DC, et al. Evaluation of potential biomarkers for the diagnosis and monitoring of Systemic Lupus Erythematosus using the Cytometric Beads Array (CBA). Clinica Chimica Acta. 2019;499:16-23.

31. Kümpers P, David S, Haubitz M, Hellpap J, Horn R, Bröcker V, et al. The Tie2 receptor antagonist angiopoietin 2 facilitates vascular inflammation in systemic lupus erythematosus. Ann Rheum Dis. 2009;68(10):1638-43.

32. Curiel RV, Bhagati R, Basavaraju L, Norton D, Katz J, Haile E, et al. Von Willebrand factor, red cell fragmentation, and disease activity in systemic lupus erythematosus. Hss j. 2008;4(2):170-4.

33. Heshmat NM, El-Kerdany TH. Serum levels of vascular endothelial growth factor in children and adolescents with systemic lupus erythematosus. Pediatr Allergy Immunol. 2007;18(4):346-53.

34. Zhou L, Lu G, Shen L, Wang L, Wang M. Serum levels of three angiogenic factors in systemic lupus erythematosus and their clinical significance. Biomed Res Int. 2014;2014:627126.

35. Boehme MW, Raeth U, Galle PR, Stremmel W, Scherbaum WA. Serum thrombomodulin-a reliable marker of disease activity in systemic lupus erythematosus (SLE): advantage over established serological parameters to indicate disease activity. Clin Exp Immunol. 2000;119(1):189-95.

36. Horák P, Scudla V, Hermanovó Z, Pospisil Z, Faltýnek L, Budiková M, et al. Clinical utility of selected disease activity markers in patients with systemic lupus erythematosus. Clin Rheumatol. 2001;20(5):337-44.

37. Mahmood A, Farhad G, Mehrzad H, Abdolhadi N, Ahmad Reza J, Farhad S, et al. Assessment of Serum Thrombomodulin in Patients with Systemic Lupus Erythematosus in Rheumatology Research Center. Acta Medica Iranica. 1970;47(2).

38. Sari RA, Taysi S, Erdem F, Yilmaz O, Keleş S, Kiziltunç A, et al. Correlation of serum levels of soluble intercellular adhesion molecule-1 with disease activity in systemic lupus erythematosus. Rheumatol Int. 2002;21(4):149-52.
